# The serine protease DPP9 and the redox sensor KEAP1 form a mutually inhibitory complex

**DOI:** 10.1016/j.jbc.2024.108034

**Published:** 2024-11-29

**Authors:** Lydia P. Tsamouri, Jeffrey C. Hsiao, Daniel A. Bachovchin

**Affiliations:** 1 Pharmacology Program of the Weill Cornell Graduate School of Medical Sciences, Memorial Sloan Kettering Cancer Center, New York, New York, USA; 2 Chemical Biology Program, Memorial Sloan Kettering Cancer Center, New York, New York, USA; 3 Tri-Institutional PhD Program in Chemical Biology, Memorial Sloan Kettering Cancer Center, New York, New York, USA

**Keywords:** DPP9, KEAP1, inhibition, NRF2, protease, inflammasome, redox, NLRP1, CARD8

## Abstract

Synthetic inhibitors of the serine protease DPP9 activate the related NLRP1 and CARD8 inflammasomes and stimulate powerful innate immune responses. Thus, it seems plausible that a biomolecule similarly inhibits DPP9 and triggers inflammasome activation during infection, but one has not yet been discovered. Here, we wanted to identify and characterize DPP9-binding proteins to potentially uncover physiologically relevant mechanisms that control DPP9’s activity. Notably, we found that the redox sensor protein KEAP1 binds to DPP9 in an inactive conformation and stabilizes this non-native fold. At the same time, this inactive form of DPP9 reciprocally inhibits the ability of KEAP1 to bind to and degrade the transcription factor NRF2, thereby inducing an antioxidant response. Although we discovered several experimental conditions, for example new protein expression and chemical denaturation, that force DPP9 out of its folded dimeric state and into a KEAP1-binding state, the key danger-related stimulus that causes this critical DPP9 conformational change is not yet known. Regardless, our data now reveal that an endogenous DPP9 inhibition mechanism does in fact exist, and moreover that DPP9, like the other NLRP1 regulator thioredoxin-1, is directly coupled to the intracellular redox potential. Overall, we expect this work will provide the foundation to discover additional biomolecules that regulate DPP9’s activity, the DPP9-KEAP1 interaction, the intracellular redox environment, and the NLRP1 and CARD8 inflammasomes.

Several pathogen- and danger-associated signals induce the formation of multiprotein complexes called inflammasomes (1, 2). Inflammasomes recruit and activate the cysteine protease caspase-1, which in turn cleaves and activates the inflammatory cytokines interleukin-1β and 18 (IL-1β/18) and the pore-forming protein gasdermin D (GSDMD), thereby triggering an inflammatory form of lytic cell death called pyroptosis. Intriguingly, synthetic small molecule inhibitors of dipeptidyl peptidase 9 (DPP9), including Val-boroPro (VbP), potently activate the related NLRP1 and CARD8 inflammasomes (Figs. 1*A* and S1*A*) (3, 4, 5, 6, 7, 8, 9). As such, it seems likely that a certain danger-related ligand or process similarly inhibits DPP9 and activates these inflammasomes (7, 10). However, a physiologically relevant (*i.e.*, non-synthetic) DPP9 inhibitor has not been identified.

**Figure 1:**
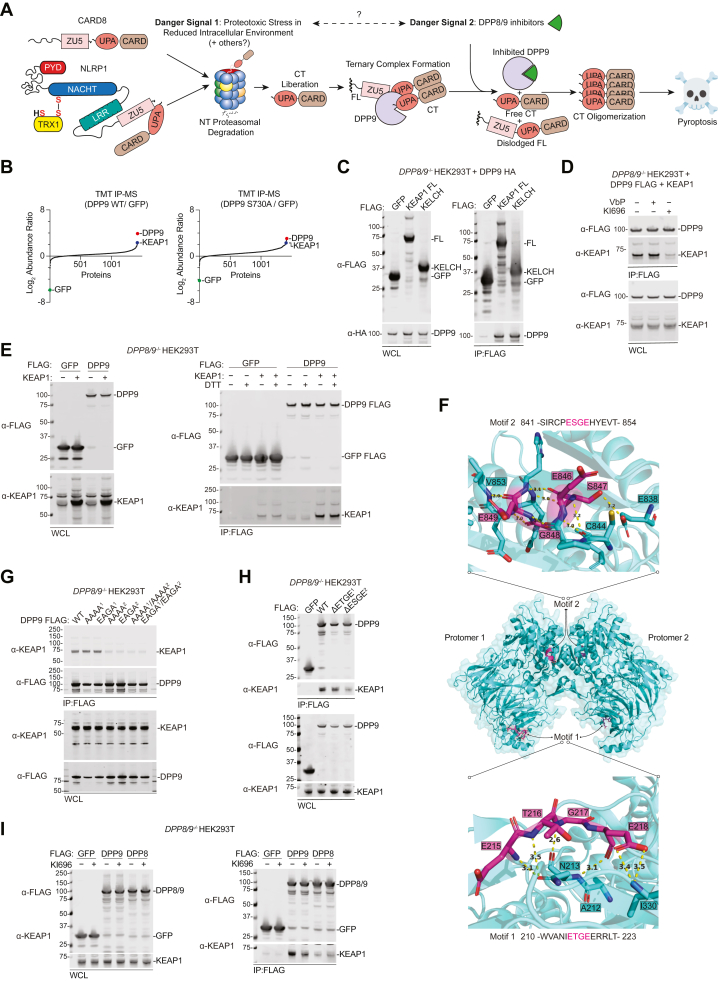
**DPP9 associates with the KEAP1 KELCH domain.***A*, schematic of NLRP1 and CARD8 inflammasome activation. *B*–*E*, the indicated proteins were transiently expressed in *DPP8/9*^*−/−*^ HEK 293T cells, immunoprecipitated (IPed) using anti-FLAG agarose beads, and analyzed by quantitative mass spectrometry (*B*) or immunoblotting *C*–*E*, in *D*, cells were treated VbP (10 μΜ) and KI696 (5 μΜ) before harvesting lysates. In *E*, the agarose beads were treated with DTT (10 mM) before elution of the FLAG-tagged proteins. *F*, crystal structure of the DPP9 dimer (6EOQ). The E(S/T)GE Motifs 1 and 2 are colored pink. *G*–*I*, the indicated FLAG-tagged DPP9 proteins were transiently expressed in *DPP8/9*^*−/−*^ HEK 293T cells, IPed using anti-FLAG agarose beads, and analyzed by immunoblotting. All immunoblots, are representative of three or more independent experiments. All immunoblots are SDS-PAGE unless indicated otherwise. HA, hemagglutinin tag; WCL, whole cell lysate.

Although this endogenous DPP9 inhibitor has remained elusive, our understanding of NLRP1 and CARD8 inflammasome activation has advanced considerably in recent years (Fig. 1*A*) (7, 10, 11, 12). Both the NLRP1 and the CARD8 proteins have function-to-find domains (FIINDs) that undergo autoproteolysis to generate non-covalently associated N-terminal (NT) and C-terminal (CT) fragments (Fig. S1*A*) (13, 14, 15). In the first activation step, the proteasome degrades the autoinhibitory NT fragment and releases the inflammatory CT fragment (Fig. 1*A*) (16, 17). Notably, a reducing intracellular environment and proteotoxic stress accelerate NT degradation (Fig. 1*A*) (18, 19, 20, 21, 22). The mechanisms controlling degradation are not yet fully established, but recent studies have revealed that the oxidized form of thioredoxin-1 binds to and stabilizes the NT of NLRP1 (18, 19, 20). The released CT fragment is then captured in a ternary complex with a full-length (*i.e.*, non-degraded) NLRP1 or CARD8 protein and DPP9. In the second activation step, synthetic (and presumably biological) DPP9 inhibitors destabilize this complex, thereby liberating CT fragments to self-oligomerize and nucleate an inflammasome (Fig. 1*A*). It seems plausible that “Danger Signal 1”—redox-linked proteotoxic stress—is in some way related to “Danger Signal 2”—DPP9 inhibition—but this connection has not yet been discovered.

DPP9 is a serine protease that releases Xaa-Pro dipeptides (where Xaa is any amino acid) from the N-termini of polypeptides (23, 24), but the critical DPP9 substrates and functions have not been definitively established (25). Studies over the last 15 years have implicated DPP9 in a variety of biological processes beyond direct NLRP1 and CARD8 repression (26, 27, 28, 29, 30) (Fig. S1*B*), but it remains unclear which, if any, of these are relevant to inflammasome regulation. First, DPP9 has been reported to play a critical role in the processing of cytoplasmic proline-containing peptides for presentation on MHC-I (26) (Fig. S1*B* part 1). However, DPP9 is not involved in the catabolism of the vast majority of such peptides (25), and thus this seems unlikely to be DPP9’s key activity. Second, DPP9 has been shown to cleave the N-terminal two amino acids from several intact intracellular proteins, including BRCA2 (28), SYK (27), and AK2 (29), thereby inducing their rapid degradation by the proteasome (Fig. S1*B* part 2). Although this mechanism couples DPP9 to proteostasis, no clear link yet exists between these proteins and the NLRP1 and CARD8 inflammasomes. Third, and perhaps most provocatively, an ESGE motif near the active-site histidine of DPP9 was recently reported to bind to the KELCH domain of KEAP1, interfering with the binding and ubiquitination NRF2 and thereby inducing an antioxidant response (Fig. S1*B* part 3, *C* and *D*) (30, 31). However, it is not known how the interaction between DPP9 and KEAP1 is regulated, how KEAP1 association impacts the catalytic activity of DPP9, and whether this process is related to inflammasome activation.

In this study, we wanted to identify and characterize physiologically relevant mechanisms that inhibit DPP9. We began by unbiasedly searching for DPP9-binding proteins, and largely corroborated recent findings that DPP9 binds to KEAP1 *via* its ESGE motif and induces NRF2 accumulation (30, 31). Intriguingly, however, we noted that this ESGE motif is buried in DPP9’s active, dimeric structure (32), and consistently found that DPP9 must undergo a substantial conformational change into an inactive state before binding KEAP1. Although we discovered experimental conditions that induce this KEAP1-binding state, including new DPP9 protein expression and chemical denaturation, the physiologically relevant danger signal that mediates the DPP9 conformational change and subsequent KEAP1 binding has not yet been identified. Nevertheless, our data now show that DPP9 and KEAP1 engage in a mutually inhibitory relationship, uncovering a key component of an endogenous (*i.e.*, non-synthetic) mechanism of DPP9 inhibition and of antioxidant signaling.

## Results

### The KEAP1 KELCH domain binds to the DPP9 ESGE motif

We reasoned that further characterizing DPP9’s interactions with other proteins would help to illuminate its key functions. As such, we first wanted to unbiasedly identify potential DPP9-binding proteins. We expressed FLAG-tagged GFP (as a control), wild-type (WT) DPP9, or catalytically inactive S730A mutant DPP9 in HEK 293T cells, harvested lysates, and performed anti-FLAG immunoprecipitations (IP) coupled with quantitative mass spectrometry (MS) analyses (Fig. 1*B* and Tables S1–S3). Several proteins were enriched in both the DPP9 WT and S730A IPs relative to the GFP control IP, including BAG2, DNAJA1, DNAJA2, KEAP1, SLC25A5, NDUFA4, and OTUD4. As the interaction with KEAP1 has been observed previously (30, 31, 33) and directly links DPP9 to the intracellular redox state (Fig. 1*A*), we decided to investigate this interaction in greater detail.

We next wanted to confirm that the KEAP1 KELCH domain binds to DPP9 (30, 31). We therefore co-transfected FLAG-tagged GFP, full-length (FL) KEAP1, or the isolated KELCH domain together with HA-tagged DPP9 in HEK 293T cells before performing anti-FLAG IPs and immunoblotting (Fig. 1*C*). This experiment corroborated that the KELCH domain interacts with DPP9. In addition, we found that the small molecule KI696, which blocks the association between the KEAP1 KELCH domain and NRF2 (34), similarly abrogates the interaction between overexpressed DPP9-FLAG and FL KEAP1 (Fig. 1*D*). In contrast, the DPP9 inhibitor VbP, which directly interacts with S730 (35), did not impact the DPP9-KEAP1 interaction (Fig. 1*D*). This result, together with our finding that S730A mutant DPP9 still binds to KEAP1 (Fig. 1*B*), strongly suggests that DPP9’s active-site serine is not part of the DPP9-KEAP1 interface. Moreover, the reducing agent dithiothreitol (DTT) similarly had no effect on the DPP9-KEAP1 interaction (Fig. 1*E*). We should note that overexpressed KEAP1 exhibits some non-specific association to the GFP-bound agarose beads, but clearly binds considerably more to the DPP9-bound beads in these IP experiments. Collectively, these data indicate that KEAP1 KELCH domain likely associates with NRF2 and DPP9 using the same surface, and that its interaction with DPP9 does not involve DPP9’s catalytic serine nor a disulfide bond.

KEAP1 is a dimer and NRF2 is a monomer, and one NRF2 monomer interacts with the two KELCH domains of the KEAP1 dimer using a high-affinity ETGE motif and a low-affinity DLG motif (Fig. S1*B* part 3*D*) (36). DPP9 is a dimer (32) (Fig. 1*F*), and, as noted previously (30, 31), each monomer has two motifs—^215^ETGE^218^ (“Motif 1”) and ^846^ESGE^849^ (“Motif 2”)—that could potentially interact with the KELCH domain (Fig. 1*F*). Motif 2, but not Motif 1, is highly conserved across vertebrates and is also present in the closely related enzyme DPP8 (Fig. S2*A*). However, only Motif 1 adopts a β-turn conformation like NRF2’s ETGE motif (Figs. 1*F* and S2*B*), although a loop (DPP9 residues T324 to I330) near Motif 1 would clash with the KELCH domain (KEAP1 residues R415 to G430) (Fig. S2*B*, top) (32). In contrast, Motif 2 is part of an α-helix completely unlike the NRF2 ETGE β-turn and is buried at the DPP9 dimer interface (Figs. 1*F* and S2*B*). Thus, both DPP9 Motifs, and especially Motif 2, would have to undergo substantial conformational changes to bind to the KEAP1 KELCH domain like NRF2’s ETGE motif.

To evaluate the importance of the DPP9 Motifs for KELCH binding, we mutated (to either AAAA and EAGA) or deleted these motifs and then expressed and purified the FLAG-tagged DPP9 variants from *DPP8/9*^*−/−*^ HEK 293T cells. Consistent with the prior reports (30, 31), we found that mutation or deletion of Motif 2, but not Motif 1, abrogated the DPP9-KEAP1 interaction (Figs. 1, *G* and *H* and S2, *C* and *D*). We should note that both mutation and deletion of Motif 1 decreased DPP9 protein expression, but these DPP9 variants clearly retained KEAP1 binding. Consistent with competitive binding with NRF2 for KEAP1, overexpression of WT, but not Motif 2 mutant, DPP9 caused NRF2 accumulation in the nucleus (Fig. S2*E*). These results strongly indicate that Motif 2, despite having a conformation seemingly incompatible with KELCH-domain binding (Fig. S2*B*), mediates the association with KEAP1.

We next investigated KEAP1’s ability to bind DPP8, which is highly similar to DPP9 and can also repress the NLRP1 and CARD8 inflammasomes (3, 32, 37). We found that overexpressed FLAG-tagged DPP8 did not interact with endogenous KEAP1 (Fig. 1*I*) nor lead to the accumulation of NRF2 in the nucleus (Fig. S2*F*). DPP8 has the same Motif 2 ESGE sequence as DPP9 (Fig. S2*A*), and therefore it is not immediately obvious why DPP8 does not bind to KEAP1. DPP8 possesses a ^242^VTRE^245^ sequence instead of DPP9’s Motif 1 ETGE sequence (Fig. S2*A*), but, as expected, this difference is not responsible for their disparate KEAP1 affinities (Fig. S2*G*). Thus, DPP9, but not DPP8, has a feature in addition to the Motif 2 ESGE tetrapeptide that is critical for KEAP1 binding.

### KEAP1 inhibits newly expressed DPP9

**Figure 2:**
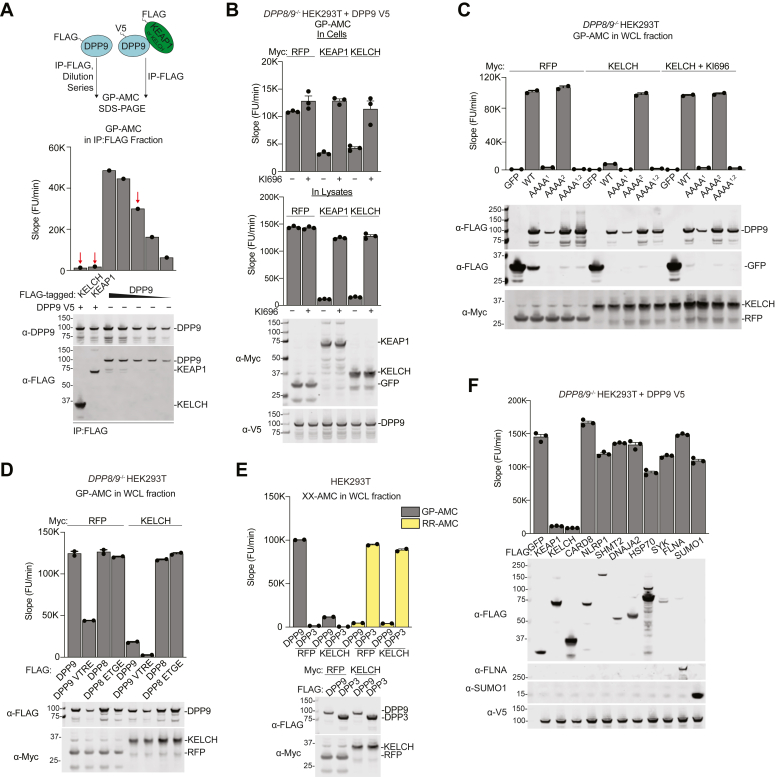
**KEAP1-bound DPP9 is catalytically inactive.***A*, FLAG-tagged DPP9 was expressed alone, and V5-tagged DPP9 was co-expressed with FLAG-tagged FL KEAP1 or the isolated KELCH domain in *DPP8/9*^*−/−*^ HEK 293T cells. The resulting lysates were subjected to anti-FLAG IP, and the protein levels and DPP9 activity in the eluates were assessed by immunoblotting and GP-AMC reporter assays, respectively. The DPP9-FLAG eluate was assessed in a five-point, two-fold dilution series to normalize the free DPP9 protein levels to the KEAP1-bound DPP9 protein levels (*red arrows* indicate comparable levels). *B–F*, the indicated WT and mutant proteins were transiently expressed in *DPP8/9*^*−/−*^ (*B–D*, and *F*) or WT (*E*) HEK 293T cells. KI696 (5 μΜ) was added to the indicated samples at the time of transfection. After 24 h, DPP9 and DPP3 activity in intact cells and/or harvested lysates (as indicated) was assessed using GP-AMC and RR-AMC assays, respectively. Harvested lysates were also analyzed by immunoblotting. GP-AMC data are means ± SEM. All data, including immunoblots, are representative of three or more independent experiments.

Although DPP9’s catalytic serine does not appear to be directly involved in the KEAP1 interaction (Fig. 1, *B* and *D*), we reasoned KEAP1 binding may nevertheless impact the enzymatic activity of DPP9. Gly-Pro-7-amido-4-methylcoumarin (GP-AMC) is a fluorogenic DPP8/9 substrate that can not only be used to measure purified DPP8/9 activity but also DPP8/9 activity in lysates and in cells (25, 26). We next overexpressed FLAG-tagged DPP9 alone in HEK 293T cells, or overexpressed V5-tagged DPP9 together with FLAG-tagged FL KEAP1 or the isolated KELCH domain (Fig. 2*A*). We then performed anti-FLAG IPs and evaluated the GP-AMC activity and DPP9 protein levels in the eluates. Strikingly, we found that the DPP9 protein that co-immunoprecipitated with FLAG-tagged FL KEAP1 or the KEAP1 KELCH domain, unlike the FLAG-tagged DPP9 protein that was directly purified, was completely inactive. In stark contrast, DPP9 protein that co-immunoprecipitated with FLAG-tagged CARD8 remained catalytically active (Fig. S3*A*).

We next wanted to explore the impact of KEAP1 binding on DPP9 activity in cells and lysates. We thus expressed DPP9 together with RFP (as a control), FL KEAP1, or the KEAP1 KELCH domain in *DPP8/9*^*−/−*^ HEK 293T cells before performing GP-AMC cleavage assays in intact cells (Fig. 2*B*, top panel) and the corresponding harvested lysates (Fig. 2*B*, bottom panel). Notably, *DPP8/9*^*−/−*^ HEK 293T have almost no detectable GP-AMC activity before ectopic DPP9 overexpression (Fig. 2*C*) (9). Similar to the results with purified proteins, we found that expression of FL KEAP1 or the isolated KELCH domain severely abrogated DPP9 activity both in cells and in lysates (Fig. 2*B*). Notably, KI696 treatment at the time of transfection completely restored DPP9 activity in these experiments, confirming that KEAP1 binding was responsible for DPP9 inhibition (Fig. 2*B*). As expected, overexpression of the KEAP1 KELCH domain did not inhibit co-expressed Motif 2 mutant DPP9 proteins, WT DPP8, or DPP8 with its VTRE sequence mutated to ETGE (Fig. 2, *C* and *D*). Several DPP9 mutants, including the Motif 1 AAAA mutant, the Motif 1 EAGA mutant, the Motif 1 deletion, and the Motif 2 deletion, were catalytically inactive due to impaired protein folding, as evidenced by a disappearance of the DPP9 dimer on Blue-Native (BN)-PAGE gels and by increased sensitivity to limited proteolysis (Figs. 2*C* and S3, *B*–*E*). Consequently, we were unable to assess the ability of KEAP1 to inhibit these particular proteoforms. However, the Motif 1 VTRE mutant (*i.e.*, to the corresponding residues in DPP8) retained at least some catalytic activity and was inhibited by KELCH overexpression (Fig. 2*D*), further showing that Motif 1 is not required for KEAP1-mediated binding and inhibition.

In contrast to these results, KELCH overexpression did not inhibit the activity of co-expressed dipeptidyl peptidase 3 (DPP3), which, like DPP9, also binds to the KEAP1 KELCH domain (Fig. 2*E*) (38). In addition, co-expression of DPP9 with other confirmed and potential binding partners, including NLRP1 and CARD8, those discovered in our proteomic experiment (*i.e.*, DNAJA2, HSP70) (Fig. 1*B* and Table S1), and those identified in the BioPlex database (33), did not appreciably inhibit DPP9 activity in harvested lysates (Fig. 2*F*). Overall, these results show that overexpressed KEAP1 and overexpressed DPP9 associate in a specific way that inhibits DPP9’s catalytic activity.

### Overexpressed KEAP1 does not inhibit preexisting DPP9

**Figure 3:**
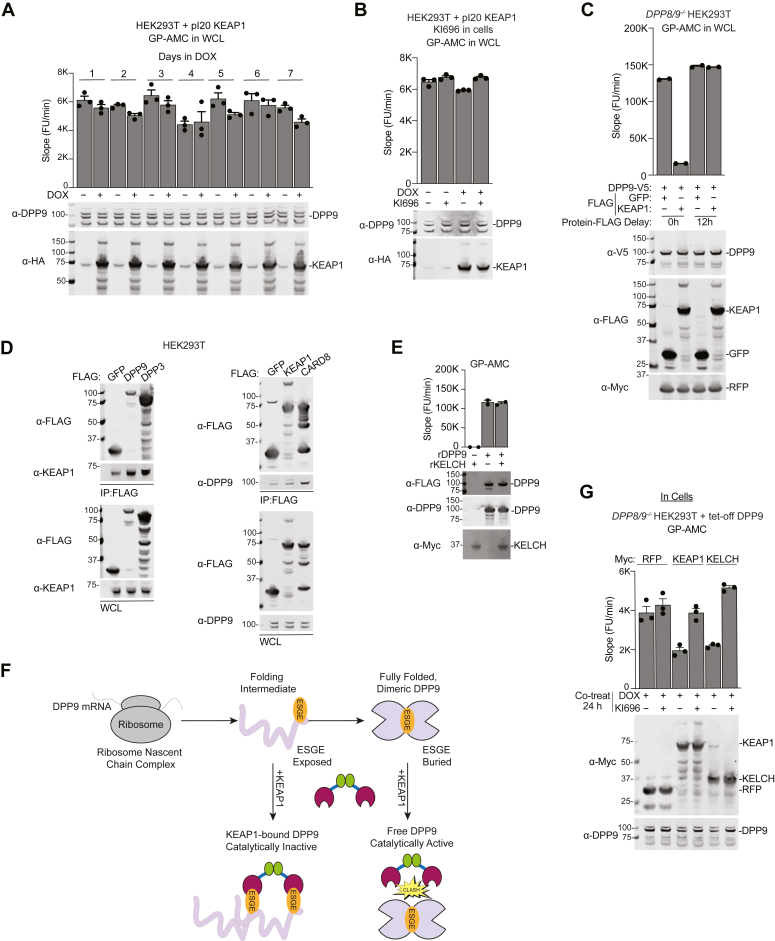
**KI696 restores KEAP1-bound DPP9 activity in cells.***A* and *B*, HEK 293T cells with a stably integrated, HA-tagged, and DOX-inducible KEAP1 construct were treated with DOX (100 ng/ml) and KI696 (5 μΜ) (*B*) for the indicated number of days in (*A*) and for 48 h in (*B*). DPP9 activity in cells and KEAP1 and DPP9 protein levels in lysates were assessed using GP-AMC reporter assays and immunoblotting, respectively. *C*, the indicated proteins were transiently expressed in *DPP8/9*^*−/−*^ HEK 293T cells. The FLAG-tagged proteins were either co-expressed with DPP9-V5 (indicated as 0 h) or transfected 12 h later (indicated as 12 h). Harvested lysates were analyzed by GP-AMC and immunoblotting assays. *D*, the indicated proteins were transiently expressed in HEK 293T cells, IPed using anti-FLAG agarose beads, and analyzed by immunoblotting. *E*, recombinant DPP9 was incubated alone or with recombinant KELCH at 1:1 ratio *in vitro* and was incubated for 2 h at 37 °C prior to GP-AMC analysis. *F*, proposed model for the KEAP1-mediated inhibition of newly expressed, but not already folded, DPP9. *G*, RFP, KEAP1, and the isolated KELCH domain were transiently expressed in *DPP8/9*^*−/−*^ HEK 293T cells with doxycycline (DOX)-off DPP9 construct. 24 h later the cells were treated with DOX (100 ng/ml) and KI696 (5 μΜ), and the next day intact cells and lysates were analyzed by GP-AMC and immunoblotting assays, respectively. GP-AMC data are means ± SEM. All data, including immunoblots, are representative of three or more independent experiments.

We should emphasize that KEAP1 and DPP9 were both ectopically overexpressed at the same time in the above experiments. We next wanted to explore the impact of KEAP1 overexpression on endogenous and/or pre-existing DPP9. To do this, we stably expressed KEAP1 in HEK 293T and in THP-1 WT cells, before evaluating the activity of endogenous DPP8/9 in harvested lysates (Figs. 3*A* and S4*A*). Notably, although both DPP8 and 9 are present in these cells, DPP9 is responsible for the vast majority of GP-AMC cleavage (9). Intriguingly, we observed that KEAP1 overexpression did not block catalytic activity of endogenous DPP9. Furthermore, KI696 treatment did not impact endogenous DPP9 activity in control and KEAP1-overexpressing HEK 293T cells (Fig. 3*B*). We next transiently transfected HEK 293T cells with cDNA encoding KEAP1 simultaneously with cDNA encoding DPP9 or 12 h following the DPP9 cDNA transfection. Strikingly, we observed that KEAP1 only inhibited DPP9 when simultaneously transfected (Fig. 3*C*). We reasoned that KEAP1 might readily bind to and maintain DPP9 in an inactive state only when co-transfected because it binds to newly synthesized DPP9 before it adopts a fully folded and dimeric structure (Fig. S2*B*). Consistent with this hypothesis, we found that overexpressed DPP9 associates with endogenous KEAP1, but that overexpressed KEAP1 does not associate with endogenous DPP9 (Fig. 3*D*). Similarly, we also found that the purified recombinant KEAP1 KELCH does not inhibit purified recombinant DPP9 (Fig. 3*E*). Overall, these data strongly suggest that fully folded DPP9 does not bind to KEAP1 (Fig. 3*F*).

We next wanted to determine if the activity of the KELCH-bound DPP9 is restored upon KELCH dissociation. We found that KI696 did not regenerate DPP9 activity when added the DPP9-KELCH complex in lysates (Fig. S4*B*), but did when added to living cells (Fig. S4*C*). The restoration of DPP9 activity in cells was not due to synthesis of new DPP9 protein, as KI696 similarly re-activated DPP9 even when DPP9 expression was repressed using a DOX-off vector (Figs. 3*G* and S4*D*). Collectively, these data indicate that overexpressed KEAP1 specifically binds to overexpressed DPP9 before it is properly folded and thereby abolishes DPP9’s catalytic activity (Fig. 3*F*), and moreover that additional cellular factors are required to fold DPP9 into an active enzyme after KEAP1 is displaced.

### Overexpressed KEAP1 binds to misfolded DPP9

**Figure 4:**
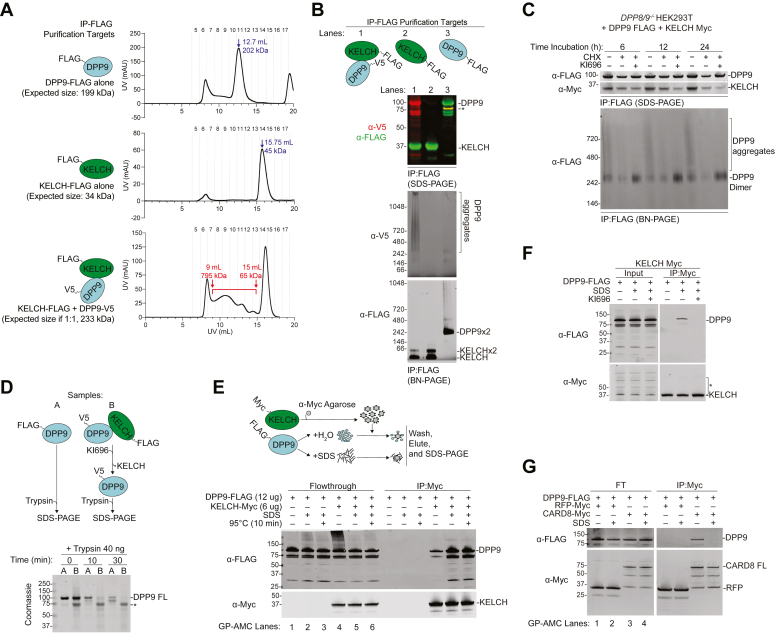
**KEAP1-bound DPP9 is misfolded.***A* and *B*, the indicated proteins were expressed alone or together in Expi293F cells, purified by anti-FLAG IP, and subjected to size exclusion chromatography (SEC) (*A*) or immunoblotting (SDS- and BN-PAGE) analyses (*B*). The UV absorption (280 nm) of each SEC fraction is shown. The *blue arrows* denote the peaks corresponding to DPP9 dimers and KELCH monomers, whereas the red bracket denotes the diffused KELCH-DPP9 complex. *C*, the indicated proteins were transiently expressed in *DPP8/9*^*−/−*^ HEK 293T cells and IPed using anti-FLAG agarose beads. CHX (50 μΜ) and KI696 (5 μΜ) were co-treated for the indicated times. *D*, purified DPP9 and DPP9 dissociated from the purified KEAP1-DPP9 complex using KI696 were subjected to trypsin-mediated limited proteolysis and analyzed by Coomassie staining. *Asterisk* denotes an unknown band. *E–G*, purified DPP9 was treated with vehicle (H_2_O) or SDS (1%) for 30 min before being diluted and loaded onto KEAP1 KELCH-domain (*E* and *F*) or CARD8 and RFP (*G*) bound agarose beads. The beads were then washed, eluted, and analyzed by immunoblotting. Flowthrough (FT) denotes the fraction that did not bind to the agarose beads. GP-AMC data for each FT fraction are found in S5G. In *F*, KI696 (50 μΜ) was added to the agarose-bound KELCH, prior to DPP9 addition. GP-AMC data are means ± SEM. All data, including immunoblots, are representative of three or more independent experiments.

We next sought to further confirm that DPP9 is not completely folded in the observed DPP9-KEAP1 complexes. As anticipated, when expressed and purified alone, DPP9-FLAG eluted as a dimer and KELCH-FLAG as a monomer (Fi. 4*A*, top and middle panel; Figures 4*B* lanes 2 and 3, S2*F*, and S5*A*). However, when DPP9-V5 and KELCH-FLAG were co-expressed and subsequently anti-FLAG purified, DPP9 and at least some of KELCH domain appeared as high-molecular-weight aggregates (Fig. 4*A*, bottom panel; Figs. 4*B*, lane 1 and S5, *B* and *C*). We observed similar results when DPP9-FLAG and KELCH-MYC were co-expressed and anti-FLAG purified (Fig. S5*D*). As expected, KI696 restored the dimeric DPP9 species in these co-overexpress 1% ion experiments (Fig. 4*C*). Moreover, DPP9 purified in association with the KELCH domain was more susceptible to trypsin-mediated proteolysis than DPP9 purified alone, consistent with an unfolded state (Fig. 4*D*). In contrast to the DPP9-KEAP1 complex, the co-expression of DPP9 with either CARD8 or NLRP1 (Fig. S5*E*), and DPP3 with KEAP1 (Fig. S5*F*) retained folded conformations and did not form high-molecular weight aggregates.

To provide additional evidence that the KELCH domain can indeed bind to misfolded or differently folded DPP9, we denatured purified DPP9 using 1% sodium dodecyl sulfate (SDS) and boiling, diluted these samples to <0.1% SDS, and then applied the protein to the immobilized KELCH domain. We observed that denatured DPP9 was completely inactive (Fig. S5*G* top) but nevertheless bound more to tightly to the KELCH domain (Fig. 4*E*). Importantly, KI696 completely abrogated the interaction between denatured DPP9 and KEAP1 (Fig. 4*F*), showing that this remains a specific association. In contrast, DPP9 binds to CARD8 in a folded state (39), and, as expected, SDS treated-DPP9 did not interact with CARD8 (Figs. 4*G* and S5*G* bottom). Collectively, these experiments indicate that DPP9 must undergo a substantial conformational change (or, in the case of co-expression, not yet have adopted a completely folded conformation) to bind to KEAP1, likely because the ESGE motif in the folded dimeric DPP9 structure is shielded from interacting with the KELCH domain (Fig. S2*B*).

### DPP9 C844 is critical for KEAP1 binding

**Figure 5:**
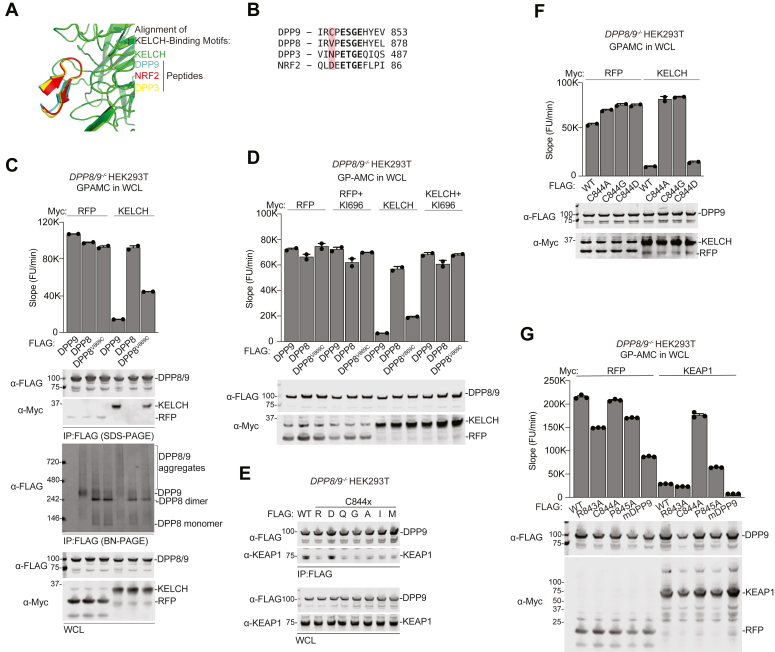
**C844 on DPP9 is required for KEAP1 binding**. *A*, overlay of the AlphaFold 3 predictions of the DPP9, NRF2, and DPP3 E(S/T)GE peptides. *B*, sequence alignment of the peptides in *A*. *C–G*, the indicated proteins were transiently expressed in *DPP8/9*^*−/−*^ HEK 293T cells. The lysates and anti-FLAG IPs were analyzed by immunoblotting (SDS- and BN-PAGE) and GP-AMC assays as indicated. GP-AMC data are means ± SEM. All data, including immunoblots, are representative of three or more independent experiments.

The abovementioned experiments suggest that only the residues in and around Motif 2, and not the tertiary structure of DPP9, are critical for KEAP1 binding. Consistent with this premise, AlphaFold 3 (40) predicts that the 12 residue region encompassing Motif 2 (^842^IRCP**ESGE**HYEV^853^) binds to the KELCH domain like the corresponding regions in NRF2 and DPP3 (Fig. 5, *A* and *B*). Intriguingly, these 12 residues in DPP8 and DPP9 are highly similar, with the notable exception that DPP8 has a valine (V869) instead of a cysteine (DPP9 C844) preceding its Motif 2 ESGE sequence (Figs. S2*A* and 5*B*). We found that mutation of DPP8 V869 to cysteine remarkably rendered DPP8 sensitive to KELCH binding, KELCH-mediated inhibition, and KELCH-mediated aggregate formation (Fig. 5, *C* and *D*), showing that this residue is a key difference between these two peptidases. Further highlighting the importance of this cysteine, we found that mutation of DPP9 C844 to R, Q, G, A, I, and M completely disrupted binding to KEAP1 (Fig. 5*E*). However, it should be noted that DPP9 C844D retained both catalytic activity and sensitivity to KEAP1-mediated inhibition (Fig. 5, *E* and *F*), thus this cysteine is not the only amino acid that can support both of these functions. Unlike C844, mutation of R843 or P845 to A retained catalytic activity and sensitivity to KEAP1 (Fig. 5*G*), suggesting that the identities of these nearby residues are not as important as C844. Consistently, P845 is not completely conserved in across vertebrates (Fig. S2*A*). For example, mouse DPP9 has an arginine in this position, and mouse DPP9 remains susceptible KEAP1-mediated inhibition (Fig. 5*G*). Overall, these data further suggest that the sequence around and including Motif 2 adopts a conformer unlike the helix in the dimeric DPP9 structure before binding to KEAP1.

### Oxidants do not induce KEAP1-DPP9 interaction

**Figure 6:**
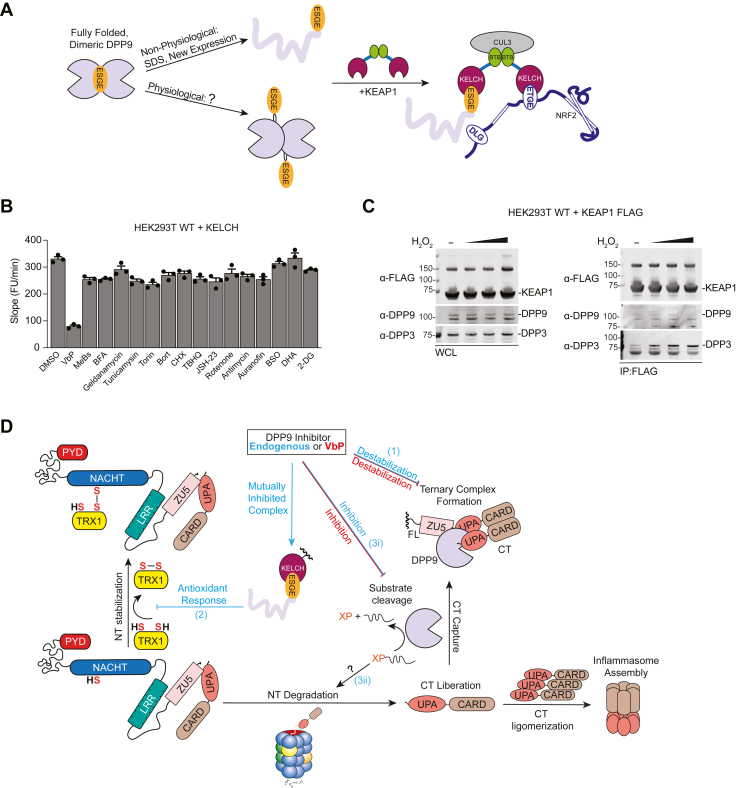
**Oxidative and proteotoxic stressors do not inhibit DPP9***A*, schematic of the proposed non-physiological (*i.e.*, new overexpression and SDS denaturation). and physiological (*i.e.*, unknown) stress-induced formation of the KEAP1-DPP9 complex. *B*, the KEAP1 KELCH domain was transiently expressed in HEK 293T cells. The next day, cells were treated with compounds for 6 h before DPP9 activity was assessed using GP-AMC assays. All compounds were used at 10 μΜ except BFA (2 μΜ), Bort (1 μΜ), CHX (100 μΜ), TBHQ (100 μΜ), BSO (1 mM), DHA (250 μΜ), 2-DG (5 mM). *C*, KEAP1-FLAG was transiently expressed in HEK 293T cells. After 48 h, cells were treated with H_2_O_2_ (100, 250, 500 μΜ) for 3 h before lysates were harvested. KEAP1-FLAG was IPed using anti-FLAG agarose beads, and the indicated fractions were analyzed by immunoblotting. GP-AMC data are means ± SEM. All data, including immunoblots, are representative of three or more independent experiments. *D*, proposed unifying mechanism for NLRP1 activation. An unknown pathogen or stress leads to the accumulation of the DPP9 inhibitor. This proposed endogenous (*i.e.*, not synthetic) inhibitor binds to and induces a conformational change on DPP9, that in turn leads to KEAP1 binding. This biomolecule and the mutually inhibitory complex can contribute to inflammasome activation in at least three ways (1). The inhibitor, like VbP, disrupts the DPP9-NLRP1 complex (2). KEAP1 inhibition drives an NRF2-mediated reductive cell state that increases the levels of reduced TRX1. (3i) Inhibition of DPP9’s catalytic activity results in the accumulation of uncleaved peptides that (3ii) cause proteotoxic stress and accelerate NT degradation.

The abovementioned experiments used experimental techniques (*e.g.*, overexpression and SDS denaturation) to force DPP9 into a conformation that binds to KEAP1. We hypothesized that an as yet unknown, physiologically relevant stimulus likely induces the necessary change in DPP9’s structure to bind to KEAP1 (Fig. 6*A*). As KEAP1 is thought to respond to acute oxidative and electrophilic stress and as DPP9 has critical cysteine at the KEAP1 interface (36), we reasoned that exogenous oxidants and/or electrophiles might modify this cysteine (or potentially other cysteines in DPP9), thereby driving the DPP9-KEAP1 interaction and stabilizing NRF2. We thus treated HEK 293T cells stably expressing the KEAP1 KELCH domain (to increase the propensity of DPP9 to bind to participate in this interaction) with a panel of known oxidants and electrophiles, including auranofin, H_2_O_2_, tert-butylhydroquinone (TBHQ), electron transport chain inhibitors (rotenone and antimycin), and dehydroascorbate (DHA). However, we found that none substantially impacted DPP9 activity in these cells (Fig. 6*B*). Moreover, even though treatment of cells with H_2_O_2_ has been reported to increase KEAP1’s affinity for both DPP9 (30) and DPP3 (41), we only observed an H_2_O_2_-induced increase in the KEAP1-DPP3 interaction (Figs. 6*C* and S6). We additionally tested several proteotoxic agents, including geldanamycin and brefeldin A, that accelerate NLRP1 and CARD8 NT degradation (22), but these agents similarly did not cause DPP9 inhibition (Fig. 6*B*). Collectively, these data indicate that commonly used oxidants, electrophiles, and proteotoxic stressors do not induce the formation of the KEAP1-DPP9 inhibitory complex.

## Discussion

Synthetic, active site-directed DPP9 inhibitors trigger the assembly of the related NLRP1 and CARD8 inflammasomes, thereby triggering pyroptotic cell death and strong downstream immune responses (Fig. 1*A*) (3, 5, 6, 7, 42). Thus, it seems highly likely that these inflammasomes evolved to detect an endogenous (non-synthetic) DPP9 inhibitor. Despite extensive investigations, however, such “physiologically relevant” inhibitors of DPP9 have not been identified (3, 25, 43, 44, 45). In this work, we sought to further illuminate the biological functions of DPP9 by identifying and characterizing DPP9-binding proteins. Our initial efforts demonstrated that *overexpressed* DPP9 binds to the KELCH domain of KEAP1 *via* its C-terminal ESGE sequence (dubbed “Motif 2”), thereby stabilizing the antioxidant transcription factor NRF2 (Fig. S2*B*, part 3). Notably, these findings largely corroborated two recent studies that first reported on the DPP9-KEAP1 association (30, 31). However, many unknowns remained regarding the DPP9-KEAP1 interaction, including the mechanisms that control the formation of this protein-protein complex.

Most notably, although DPP9 Motif 2 clearly mediates the interaction with KEAP1 (Fig. 1, *G* and *H*), these residues are buried at the DPP9 dimer interface in a conformation seemingly incompatible with KELCH binding in all apo-, inhibitor-bound, and NLRP1/CARD8-bound structures (Figs. 1*F* and S2*B*) (32, 35, 46, 47). Thus, we speculated that DPP9 must adopt a conformation dramatically different from those previously observed prior to KEAP1 binding. Consistent with this idea, we found that the overexpressed DPP9 bound to overexpressed KEAP1 was not dimeric. Instead, the KEAP1 KELCH domain trapped nascent DPP9 before it had completed folding (Fig. 3*D*). Consistently, we found that the KEAP1 KELCH domain did not appreciably bind to and inhibit already folded dimeric DPP9. Thus, we conclude that KEAP1 binds to DPP9 in a conformation unlike any seen previously, and that DPP9 passes through a state close enough to this conformation during folding that KEAP1 can associate with it. However, we should emphasize that it is unlikely that KEAP1 acts to inhibit newly expressed DPP9 in native biological systems, as endogenous KEAP1 does not inhibit endogenous DPP9 in otherwise unstressed cells. Alternatively, we hypothesize that a stimulus likely induces a specific conformational change in DPP9 that forces Motif 2 into the KEAP1-binding conformation (Fig. 6*A*).

What might this “unknown stimulus” be? Here, we considered the possibility that oxidative or electrophilic stress acts directly on DPP9, triggering its interaction with KEAP1 and the expression of antioxidant genes to resolve this stress. Consistent with this idea, we discovered that a cysteine residue in DPP9 (C844) near Motif 2 is critical for KEAP1 binding, providing a plausible site of redox regulation. However, we found that commonly used oxidizing agents, reducing agents, and electrophiles did not appreciably impact the DPP9-KEAP1 interaction (Figs. 1*E* and 6*B*). Thus, it appears that oxidative stress is not the key stimulus that brings these proteins together. We also reasoned that proteotoxic stress might induce DPP9 to unfold in a way that enables KEAP1 binding, but commonly used proteotoxic stressors similarly did not trigger this interaction (Fig. 6*B*).

An alternative possibility is that some specific biomolecule (*e.g.*, a ligand, metabolite, or peptide) that is in some way related to metabolism or redox biology accumulates in the cytosol during a certain danger state and binds to DPP9 (Fig. 6*D*). This binding event, perhaps occurring near the DPP9 active-site and Motif 2, could potentially force DPP9 into the inactive conformation that associates with KEAP1. We should emphasize that this binding event would likely contribute to inflammasome activation in at least three ways. First, the biomolecule, like VbP, would disrupt the DPP9-NLRP1 complex. Second, the resulting KEAP1 inhibition would drive an NRF2-mediated reductive cell state and the dissociation of oxidized TRX1 from NLRP1. Third, the inhibition of DPP9’s catalytic activity would cause to the accumulation of certain uncleaved peptides that cause proteotoxic stress and thereby accelerate NT degradation or in some other way impact inflammasome assembly. We should note that VbP elicits two of these three responses, but lacks the ability to induce KEAP1 binding and therefore may not activate the NLRP1 inflammasome as powerfully as the proposed endogenous DPP9 inhibitor.

In conclusion, our work here demonstrates that DPP9 and KEAP1 engage in a mutually inhibitory relationship and that this interaction is likely induced by a specific, but as yet unknown, biomolecule. Based on these findings, we now propose that the seemingly disparate signals that activate the NLRP1 inflammasome—the accelerated degradation of unfolded proteins, the reduction of the intracellular redox potential, and the destabilization of the DPP9-NLRP1 ternary complex—are all directly caused by the appearance of this “danger signal” (Fig. 6*D*). We anticipate that the successful identification of this dangerous biomolecule will provide a breakthrough not only in our understanding of innate immunity, but also of the basic mechanisms that control cell metabolism, the intracellular oxidation state, and cytosolic protein stability.

## Experimental procedures

### Antibodies and reagents

Antibodies used include: DPP9 rabbit polyclonal antibody (Abcam, ab42080), KEAP1 rabbit polyclonal antibody (Proteintech, 10503-2-AP), Myc-Tag rabbit monoclonal antibody (71D1O, Cell Signaling Technology), NRF2 rabbit polyclonal antibody (Abcam, ab137550), DPP3 rabbit polyclonal (Proteintech, 10650-1-AP), β-Tubulin rabbit monoclonal antibody (Cell Signaling, 2128), Lamin B1 rabbit polyclonal antibody (Proteintech, 12987-1-AP), FLAG mouse monoclonal Ab (Sigma Aldrich, F1804), HA mouse monoclonal Ab (Cell Signaling Tech, 6E2), V5 mouse monoclonal antibody (Sigma, V8012), IRDye 680RD Streptavidin (LICOR, 926-68079) IRDye 800CW donkey anti-rabbit (LICOR, 925-32211), IRDye 680RD donkey anti-rabbit (925-68073), IRDye 800CW donkey anti-mouse (925-32212), IRDye 680RD donkey anti-mouse (925-68072). All primary antibodies were used at 1:1000 dilution, while all secondary antibodies at 1:10,000.

Reagents for immunoprecipitation experiments include anti-FLAG M2 agarose resin (Millipore, A2220), anti-c-Myc agarose (20169, Thermo Fischer Scientific). Other reagents used include Val-boroPro (VbP; Tocris 3719), bortezomib (Bort; Millipore-Sigma, 504314, Doxycycline hyclate (Cayman, 14422), DL-Dithiothreitol (D9779, Sigma-Aldrich), Doxycycline hyclate (Cayman, 14422), bestatin methyl ester (MeBs; Sigma, 200485), Geldanamycin (GA; Santa Cruz, sc-200617), Brefeldin A (BFA; Biologend, 420601), Tunicamysin (Cayman, 11445), Torin 1 (ThermoFisher Scientific, 4247) Rotenone (ApexBio, B5462), Antimycin A (Sigma Aldrich, A8674), Auranofin (Cayman, 15316), L-Buthionine-(S,R)-Sulfoximine (BSO; Cayman, 14484), (L)-Dehydroascorbic acid (DHA; Sigma Aldrich, 261556), 2-Deoxy-D-glucose (2-DG, Sigma Aldrich, D8375), Hydrogen Peroxide Solution (H_2_O_2_, Sigma Aldrich, 95321), Diamide (Sigma Aldrich, D3648), KI696 (MedChemExpress, HY-101140), tert-butylhydroquinone (TBHQ; Sigma, 112941), FuGENE HD (Promega, E2311), HALT protease inhibitor cocktail (Thermo Scientific, 78430), PEI-STAR (7854, Tocris), Valproic Acid Sodium Salt (P4543, Sigma-Aldrich), D-Glucose (310816, Sigma-Aldrich), Sodium dodecyl sulfate (SDS; Invitrogen, AM9820), SimplyBlue Safe Stain (ThermoFisher Scientific, LC6065), Sequencing grade modified trypsin (Promega, V5113), Gly-Pro-7-amido-4-methylcoumarin (GP-AMC; Cayman, 34458), H-Arg-Arg-AMC hydrochloride salt (RR-AMC; Biosynth, FA110477), TMTsixplex Isobaric Label Reagents (ThermoFisher Scientific, 90061).

### Cell culture

HEK293T and THP-1 cells were purchased from ATCC. HEK293T *DPP8* and *DPP9* double knockout cells were generated previously (35). Expi293F cells were purchased from Thermo Fisher Scientific. HEK 293T cells were grown in Dulbecco’s Modified Eagle’s Medium (DMEM) with L-glutamine and 10% fetal bovine serum (FBS). THP-1 cells were grown in Roswell Park Memorial Institute (RPMI) medium 1640 with L-glutamine and 10% FBS. Expi293F cells were grown in Expi293 Expression Medium (A1435101, Gibco). All cells except Expi293F cells were in a 5% CO_2_ atmosphere incubator. Expi293F cells are grown at 8% CO2. All cells were grown at 37 °C. Cell lines were regularly tested for *mycoplasma* using the MycoAlert *Mycoplasma* Detection Kit (Lonza).

### Cloning

The DPP9 open reading frame (ORF) was purchased from Origene, the KEAP1 ORF was purchased from Addgene (pDONR223_KEAP1_WT, #81925), the mouse DPP9 was purchased from GenScript (OMU22860), DPP3 was purchased from GenScript (OHu26674). Site-directed mutagenesis was performed using the QuikChange II Site-directed mutagenesis kit (Agilent, 200523) according to the manufacturer’s instructions. For constitutive expressions, the indicated DPP9 mutants and KEAP1 were shuttled using Gateway technology into pLEX307 vectors that have been modified to contain various epitope tags as indicated. For the generation of tetracycline (tet)-on-inducible constructs, the Gateway-compatible pINDUCER20 plasmid was used. The tet-off vector was created by swapping out the rtTA with tTA on the pcw57.1 tet-on vector.

### Stable line generation

The indicated expression plasmids were packaged into lentivirus in HEK 293T cells as described above. 1 × 10^6^ cells of HEK293T or 2 × 10^6^ cells of THP-1 were then infected with the virus, and after 48h, selected with puromycin (0.5 μg/ml) or geneticin (200 μg/ml) until control cells died.

### Transient transfections

HEK 293T cells were plated in 12-well culture plates at 0.25 × 10^5^ cells/well in DMEM. The next day, the indicated plasmids were added to a total of 1.0 μg DNA (with pLEX307 RFP or GFP as the filler plasmid) in 65 μl Opti-MEM and transfected using FuGENE HD (Promega) according to the manufacturer’s protocol. 1 μg of each plasmid construct was used. The cells were incubated for an additional 48 h before their harvest. For Expi293F cells, cells were grown in flasks at 3 million/ml with 200 to 400 ml of the Expi293F media. For 400 ml of media, 2133 uL of PEI-STAR (1 mg/ml) was added to 40 ml of Opti-MEM containing 400 ug of DNA plasmid. The master mix was then pipetted into the flask.

### Immunoprecipitation (IP)

For IP experiments, HEK 293T cells were transiently transfected as described above. The cells were first treated with the indicated compounds in the relevant experiments. After the indicated incubation periods, cells were harvested, and the pellets were sonicated and centrifuged at 1000*g* for 5 min. The clarified lysates were then incubated with 40 μl of anti-FLAG-M2 (Sigma-Aldrich) or anti-Myc (Thermo Fisher Pierce) agarose resin for 1 h at 4 °C. After washing with three rounds of 100 μl of PBS, bound proteins were eluted by incubating resin with 100 μl of PBS containing 150 ng/μl 3X-FLAG peptide (Sigma-Aldrich) or 1 μg/ml MYC peptide (Thermo Fisher) for 1 h at room temperature.

### Sodium dodecyl sulfate (SDS)-mediated protein denaturation assays

The indicated Myc-tagged proteins were immobilized on MYC agarose beads. KI696 (50 μM) was treated to the relevant samples at this stage. Next, recombinant DPP9 was denatured with SDS (1%) for 30 min and then diluted to 0.1% with 1% IGEPAL-containing Tris Buffered Saline (TBS). The diluted DPP9 was then applied to the KEAP1 KELCH-bound agarose beads for 3 h at 25 °C. The beads are then washed and eluted with Myc peptide.

### Limited proteolysis assays

The indicated proteins were expressed in HEK 239Ts and purified with anti-FLAG agarose. Trypsin was then added at the indicated concentrations to the eluates for 20 min before the reactions were quenched with 2X loading dye and analyzed by SDS-PAGE. KI696 (100 uM) was added where indicated to dissociate DPP9 from KEAP1 KELCH-FLAG bound to the agarose beads.

### Purification of recombinant proteins and size exclusion chromatography (SEC)

Recombinant proteins were purified as previously described (35). Briefly, Expi293F cells were transfected with the indicated DNA constructs as indicated. Cells were first washed with PBS, subsequently reconstituted in buffer containing 25 mM Tris-HCl,150 mM NaCl, 1 mM TCEP, pH 7.5., sonicated (Branson, 40% amplitude, 2.5 min ON time), and centrifuged at 15,000*g*. Supernatant was then incubated with either anti-FLAG agarose resin (Sigma Aldrich) or anti-MYC agarose resin (Thermo Fisher). After 4 hours of incubation at 4 °C, the agarose beads were washed with fifty column volumes, and incubated with either 1 column volume of 3XFLAG peptide (150 ng/uL) or 1 column volume of MYC peptide (2.5 mg/ml). The eluate was then concentrated with a 10 kDa molecular weight cutoff filter (Amicon Ultra) down to 0.5 ml and injected into a Superdex 200 increase 10/300 Gl column (Cytiva).

### Immunoblotting

For SDS-PAGE, samples were loaded with 2X loading dye (Li-Cor) containing 50 mM DTT (Thermo Fisher). Protein concentrations were determined and normalized using the DCA Protein Assay kit (Bio-Rad). After samples were separated by 4 to 12% Bis-TRIS SDS gels (Thermo Fisher), denatured proteins were transferred to nitrocellulose membranes with the Trans-Blot Turbo Transfer System (BIO-RAD) for 7 min at 2.5A, up to 25V. Blue Native-PAGE was performed as previously described (48). Briefly, anti-FLAG or anti-Myc agarose purified proteins were combined 1:1 with a master mix containing 4X NativePAGE Sample Buffer (Thermo Fisher), H_2_O, and G-250 Coomassie Dye. The final concentration of Coomassie in each sample was 0.25%. The samples were then separated on a 4 to 16% NativePage gel (Thermo Fisher), first with dark blue cathode buffer (0.02% G-250) for 30 min at 150 V, then with light blue cathode buffer (0.002% G-250) for another 30′ at 300 V. The proteins were then transferred onto methanol-activated PVDF membranes with the Trans-Blot Turbo Transfer System (BIO-RAD) for 10 min at 2.5A, up to 25V. The membranes were then de-stained with buffer containing 50% methanol, 10% acetic acid, and water. The de-stained membrane was then blocked with blocking buffer, and incubated with primary antibodies overnight. Membranes were blocked with Intercept (TBS) Blocking Buffer (LI-COR) for 30 min at ambient temperature, prior to incubation with primary antibodies overnight at 4°C. Blots were washed with TBS with 0.1% Tween-20 buffer prior to incubation with secondary antibodies for 60 min at ambient temperature. Blots were visualized using the Odyssey Imaging System (Li-Cor).

### AMC substrate assays

For in cell AMC reporter assays, 1.0 × 10^5^ HEK 293T cells were seeded per well in DMEM. 30 min prior to GP-AMC addition, the cells were treated with the DPP4 inhibitor sitagliptin (50 μΜ). GP-AMC (250 μM) was then added to the media to initiate the reaction. Substrate cleavage was measured as increasing fluorescence signal (Ex/Em: 380/460 nm) recorded at 25 °C for 25 to 40 min. Cleavage rates are reported as the slope of the linear regression of AMC fluorescence vs. time data curve. For lysate (WCL) AMC reporter assays, approximately 15 μg of total lysate was used and 100 μΜ GP-AMC or RR-AMC. Assays were performed in 96- or 384-well, black, clear bottom plates (Corning). Fluorescence was recorded using either Cytation five Cell Imaging Multi-Mode Reader 500 (BioTek) or Synergy H1 Multimode Microplate Reader (BioTek).

### Statistical analysis

Two-sided Student’s *t* tests were used for significance testing. *p* values less than 0.05 were considered to be significant. Graphs and error bars represent means ± SEM of three independent experiments unless stated otherwise. The investigators were not blinded in all experiments. All statistical analysis was performed using GraphPad Prism 9.

## Data availability

All data in this study are available within the paper, supporting information, and/or from the corresponding author upon request.

## Supporting information

This article contains supporting information.

## Conflict of interests

The authors declare that they have no conflicts of interest with the contents of this article.

## Supporting information

Supp figures and legends

## References

[1] Broz P., Dixit V.M. (2016). Inflammasomes: mechanism of assembly, regulation and signalling. Nat. Rev. Immunol..

[2] Rathinam V.A., Fitzgerald K.A. (2016). Inflammasome complexes: emerging mechanisms and effector functions. Cell.

[3] Okondo M.C., Johnson D.C., Sridharan R., Go E.B., Chui A.J., Wang M.S. (2017). DPP8 and DPP9 inhibition induces pro-caspase-1-dependent monocyte and macrophage pyroptosis. Nat. Chem. Biol..

[4] Okondo M.C., Rao S.D., Taabazuing C.Y., Chui A.J., Poplawski S.E., Johnson D.C. (2018). Inhibition of Dpp8/9 activates the Nlrp1b inflammasome. Cell Chem. Biol..

[5] Johnson D.C., Taabazuing C.Y., Okondo M.C., Chui A.J., Rao S.D., Brown F.C. (2018). DPP8/DPP9 inhibitor-induced pyroptosis for treatment of acute myeloid leukemia. Nat. Med..

[6] Zhong F.L., Robinson K., Teo D.E.T., Tan K.Y., Lim C., Harapas C.R. (2018). Human DPP9 represses NLRP1 inflammasome and protects against autoinflammatory diseases *via* both peptidase activity and FIIND domain binding. J. Biol. Chem..

[7] Taabazuing C.Y., Griswold A.R., Bachovchin D.A. (2020). The NLRP1 and CARD8 inflammasomes. Immunol. Rev..

[8] Griswold A.R., Cifani P., Rao S.D., Axelrod A.J., Miele M.M., Hendrickson R.C. (2019). A chemical btrategy for protease substrate profiling. Cell Chem. Biol..

[9] Griswold A.R., Ball D.P., Bhattacharjee A., Chui A.J., Rao S.D., Taabazuing C.Y. (2019). DPP9's enzymatic activity and not its binding to CARD8 inhibits inflammasome activation. ACS Chem. Biol..

[10] Bachovchin D.A. (2021). NLRP1: a jack of all trades, or a master of one?. Mol. Cell.

[11] Yap J.K., Emming S., Schroder K. (2024). Oxidized thioredoxin 1 places a leash on NLRP1 inflammasome activity. Immunol. Cell Biol..

[12] Mitchell P.S., Sandstrom A., Vance R.E. (2019). The NLRP1 inflammasome: new mechanistic insights and unresolved mysteries. Curr. Opin. Immunol..

[13] D'Osualdo A., Weichenberger C.X., Wagner R.N., Godzik A., Wooley J., Reed J.C. (2011). CARD8 and NLRP1 undergo autoproteolytic processing through a ZU5-like domain. PLoS One.

[14] Finger J.N., Lich J.D., Dare L.C., Cook M.N., Brown K.K., Duraiswami C. (2012). Autolytic proteolysis within the function to find domain (FIIND) is required for NLRP1 inflammasome activity. J. Biol. Chem..

[15] Frew B.C., Joag V.R., Mogridge J. (2012). Proteolytic processing of Nlrp1b is required for inflammasome activity. PLoS Pathog..

[16] Chui A.J., Okondo M.C., Rao S.D., Gai K., Griswold A.R., Johnson D.C. (2019). N-terminal degradation activates the NLRP1B inflammasome. Science.

[17] Sandstrom A., Mitchell P.S., Goers L., Mu E.W., Lesser C.F., Vance R.E. (2019). Functional degradation: a mechanism of NLRP1 inflammasome activation by diverse pathogen enzymes. Science.

[18] Ball D.P., Tsamouri L.P., Wang A.E., Huang H.C., Warren C.D., Wang Q. (2022). Oxidized thioredoxin-1 restrains the NLRP1 inflammasome. Sci. Immunol..

[19] Zhang Z., Shibata T., Fujimura A., Kitaura J., Miyake K., Ohto U. (2023). Structural basis for thioredoxin-mediated suppression of NLRP1 inflammasome. Nature.

[20] Geeson M.B., Hsiao J.C., Tsamouri L.P., Ball D.P., Bachovchin D.A. (2023). The interaction between NLRP1 and oxidized TRX1 involves a transient disulfide bond. Cell Chem. Biol..

[21] Chui A.J., Griswold A.R., Taabazuing C.Y., Orth E.L., Gai K., Rao S.D. (2020). Activation of the CARD8 inflammasome requires a cisordered region. Cell Rep..

[22] Orth-He E.L., Huang H.C., Rao S.D., Wang Q., Chen Q., O'Mara C.M. (2023). Protein folding stress potentiates NLRP1 and CARD8 inflammasome activation. Cell Rep..

[23] Bjelke J.R., Christensen J., Nielsen P.F., Branner S., Kanstrup A.B., Wagtmann N. (2006). Dipeptidyl peptidases 8 and 9: specificity and molecular characterization compared with dipeptidyl peptidase IV. Biochem. J..

[24] Tang H.K., Tang H.Y., Hsu S.C., Chu Y.R., Chien C.H., Shu C.H. (2009). Biochemical properties and expression profile of human prolyl dipeptidase DPP9. Arch. Biochem. Biophys..

[25] Bhattacharjee A., Bachovchin D.A. (2023). DPP8/9 are not required to cleave most proline-containing peptides. Isr. J. Chem..

[26] Geiss-Friedlander R., Parmentier N., Moller U., Urlaub H., Van den Eynde B.J., Melchior F. (2009). The cytoplasmic peptidase DPP9 is rate-limiting for degradation of proline-containing peptides. J. Biol. Chem..

[27] Justa-Schuch D., Silva-Garcia M., Pilla E., Engelke M., Kilisch M., Lenz C. (2016). DPP9 is a novel component of the N-end rule pathway targeting the tyrosine kinase Syk. Elife.

[28] Bolgi O., Silva-Garcia M., Ross B., Pilla E., Kari V., Killisch M. (2022). Dipeptidyl peptidase 9 triggers BRCA2 degradation and promotes DNA damage repair. EMBO Rep..

[29] Finger Y., Habich M., Gerlich S., Urbanczyk S., van de Logt E., Koch J. (2020). Proteasomal degradation induced by DPP9-mediated processing competes with mitochondrial protein import. EMBO J..

[30] Chang K., Chen Y., Zhang X., Zhang W., Xu N., Zeng B. (2023). DPP9 stabilizes NRF2 to suppress ferroptosis and induce sorafenib resistance in clear cell renal cell carcinoma. Cancer Res..

[31] Zhou Y., Chen Y., Xuan C., Li X., Tan Y., Yang M. (2024). DPP9 regulates NQO1 and ROS to promote resistance to chemotherapy in liver cancer cells. Redox Biol..

[32] Ross B., Krapp S., Augustin M., Kierfersauer R., Arciniega M., Geiss-Friedlander R. (2018). Structures and mechanism of dipeptidyl peptidases 8 and 9, important players in cellular homeostasis and cancer. Proc. Natl. Acad. Sci. U. S. A..

[33] Huttlin E.L., Bruckner R.J., Navarrete-Perea J., Cannon J.R., Baltier K., Gebreab F. (2021). Dual proteome-scale networks reveal cell-specific remodeling of the human interactome. Cell.

[34] Davies T.G., Wixted W.E., Coyle J.E., Griffiths-Jones C., Hearn K., McMenamin R. (2016). Monoacidic inhibitors of the Kelch-like ECH-associated protein 1: nuclear factor erythroid 2-related factor 2 (KEAP1:NRF2) protein-protein interaction with high cell potency identified by fragment-based discovery. J. Med. Chem..

[35] Hollingsworth L.R., Sharif H., Griswold A.R., Fontana P., Mintseris J., Dagbay K.B. (2021). DPP9 sequesters the C terminus of NLRP1 to repress inflammasome activation. Nature.

[36] Baird L., Yamamoto M. (2020). The molecular mechanisms regulating the KEAP1-NRF2 pathway. Mol. Cell Biol..

[37] Van Goethem S., Van der Veken P., Dubois V., Soroka A., Lambeir A.M., Chen X. (2008). Inhibitors of dipeptidyl peptidase 8 and dipeptidyl peptidase 9. Part 2: isoindoline containing inhibitors. Bioorg. Med. Chem. Lett..

[38] Hast B.E., Goldfarb D., Mulvaney K.M., Hast M.A., Siesser P.F., Yan F. (2013). Proteomic analysis of ubiquitin ligase KEAP1 reveals associated proteins that inhibit NRF2 ubiquitination. Cancer Res..

[39] Sharif H., Hollingsworth L.R., Griswold A.R., Hsiao J.C., Wang Q., Bachovchin D.A. (2021). Dipeptidyl peptidase 9 sets a threshold for CARD8 inflammasome formation by sequestering its active C-terminal fragment. Immunity.

[40] Abramson J., Adler J., Dunger J., Evans R., Green T., Pritzel A. (2024). Accurate structure prediction of biomolecular interactions with AlphaFold 3. Nature.

[41] Lu K., Alcivar A.L., Ma J., Foo T.K., Zywea S., Mahdi A. (2017). NRF2 Induction supporting breast cancer cell survival is enabled by oxidative stress-induced DPP3-KEAP1 interaction. Cancer Res..

[42] Johnson D.C., Okondo M.C., Orth E.L., Rao S.D., Huang H.C., Ball D.P. (2020). DPP8/9 inhibitors activate the CARD8 inflammasome in resting lymphocytes. Cell Death Dis..

[43] Lankas G.R., Leiting B., Roy R.S., Eiermann G.J., Beconi M.G., Biftu T. (2005). Dipeptidyl peptidase IV inhibition for the treatment of type 2 diabetes: potential importance of selectivity over dipeptidyl peptidases 8 and 9. Diabetes.

[44] Rosenblum J.S., Kozarich J.W. (2003). Prolyl peptidases: a serine protease subfamily with high potential for drug discovery. Curr. Opin. Chem. Biol..

[45] Zolg S., Donzelli L., Geiss-Friedlander R. (2024). N-terminal processing by dipeptidyl peptidase 9: cut and Go. Biochimie.

[46] Huang M., Zhang X., Toh G.A., Gong Q., Wang J., Han Z. (2021). Structural and biochemical mechanisms of NLRP1 inhibition by DPP9. Nature.

[47] Carvalho L.A.R., Ross B., Fehr L., Bolgi O., Wohrle S., Lum K.M. (2022). Chemoproteomics-enabled identification of 4-Oxo-beta-Lactams as inhibitors of dipeptidyl peptidases 8 and 9. Angew. Chem. Int. Ed. Engl..

[48] Ahn H.K., Lin X., Olave-Achury A.C., Derevnina L., Contreras M.P., Kourelis J. (2023). Effector-dependent activation and oligomerization of plant NRC class helper NLRs by sensor NLR immune receptors Rpi-amr3 and Rpi-amr1. EMBO J..

