# Supplementary material for: The serine protease DPP9 and the redox sensor KEAP1 form a mutually inhibitory complex[image]
Source: J Biol Chem. 2024 Nov 29;301(1):108034. doi: 10.1016/j.jbc.2024.108034 (PMC11773481; doi:10.1016/j.jbc.2024.108034)
Supplement: Supp figures and legends [file mmc1.pdf]

The Serine Protease DPP9 and the Redox Sensor KEAP1 Form a Mutually Inhibitory Complex

Lydia P. Tsamouri[‡], Jeffrey C. Hsiao[‡], and Daniel A. Bachovchin[*]

[‡] These authors contributed equally.

*Correspondence to Daniel A. Bachovchin: bachovcd@mskcc.org

This PDF file includes:

Figures S1 to S6 and Table S1

Figures Legends for S1 to S6 and Table S1

S1

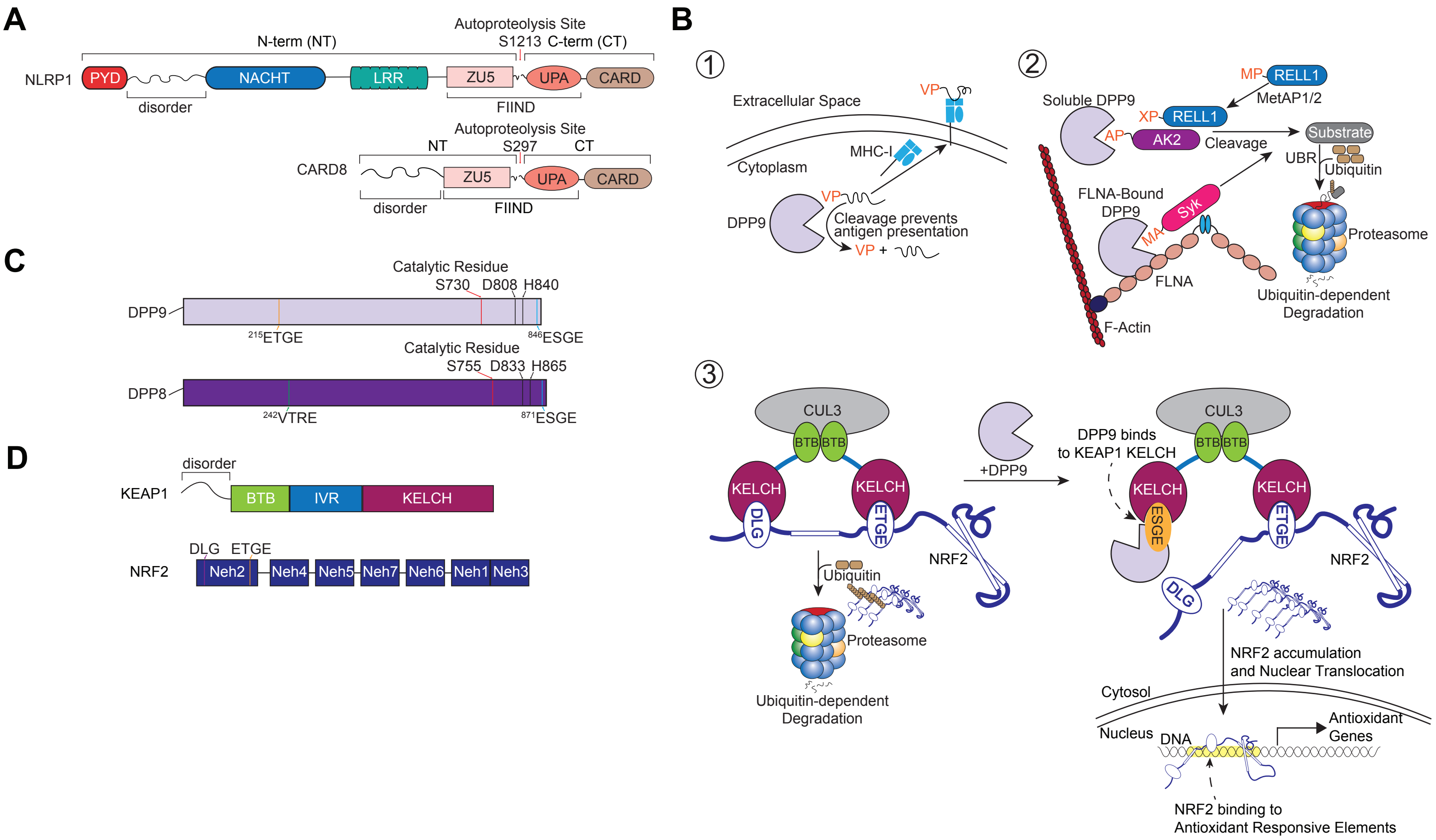
A
Autoproteolysis Site
N-term (NT)
S1213
C-term (CT)
NLRP1
PYD
NACHT
LRR
ZU5
UPA
CARD
disorder
FIIND
Autoproteolysis Site
NT
S297
CT
CARD8
ZU5
UPA
CARD
disorder
FIIND
C
Catalytic Residue
S730
D808
H840
DPP9
215ETGE
846ESGE
Catalytic Residue
S755
D833
H865
DPP8
242VTRE
871ESGE
D
disorder
KEAP1
BTB
IVR
KELCH
DLG
ETGE
NRF2
Neh2
Neh4
Neh5
Neh7
Neh6
Neh1
Neh3
B
Extracellular Space
VP
MHC-I
Cytoplasm
VP
DPP9
Cleavage prevents antigen presentation
VP +
MP
RELL1
MetAP1/2
Soluble DPP9
XP
RELL1
AP
AK2
Cleavage
Substrate
UBR
Ubiquitin
FLNA-Bound DPP9
Syk
MA
Proteasome
FLNA
Ubiquitin-dependent Degradation
F-Actin
CUL3
BTB
BTB
KELCH
KELCH
DLG
ETGE
NRF2
Ubiquitin
Proteasome
Ubiquitin-dependent Degradation
+DPP9
DPP9 binds to KEAP1 KELCH
CUL3
BTB
BTB
KELCH
KELCH
ESGE
ETGE
DLG
NRF2
NRF2 accumulation and Nuclear Translocation
Cytosol
Nucleus
DNA
Antioxidant Genes
NRF2 binding to Antioxidant Responsive Elements

Fig S1. Overview of known DPP8/9, NLRP1, and CARD8 biology. (**A**) Schematic of the NLRP1 and CARD8 proteins. (**B**) Summary of potential DPP9 bioactivities: 1) Cleaving Xaa-Pro containing peptides to prevent MHC-1 presentation, 2) cleaving N-termini of cytosolic proteins, resulting in their rapid degradation by the proteasome, and 3) binding to KEAP1 and stabilizing of the antioxidant transcription factor NRF2. (**C**,**D**) Schematic of the DPP8, DPP9, KEAP1, and NRF2 proteins. The positions of the key E(S/T)GE and catalytic residues are shown.

S2

A

DPP9 Multiple Sequence Alignment

```
Sea_lamprey            VHSGDLWVTNIRTGQELRLTHT 284
Western_clawed_frog    INNSDLWIENIHTMEEQRLTYC 193
Humans                 INNSDLWVANIETGEERRLTFC 225
Rhesus_macaque         INNSDLWVANIETGEERRLTFC 225
House_mouse            INNSDLWVANIETGEERRLTFC 195
Common_wall_lizard     INNNDLWVASIETGEERRLTYC 219
Green_sea_turtle       INNNDLWVANIETGEERRMTYC 224
Chicken                INNNDLWVANIETGEEKRMTYC 223
Saltwater_crocodile    INNNDLWVANIETGEERRMTYC 224
Monterrey_platyfish    INNNDLWIANIKTGEERRLTYC 220
Zebrafish              INNNDLWVTNIETAEERRLTFC 220
Electric_eel           INNNDLWVTNIETCEERRLTFC 220
Channel_catfish        VNNNDLWVTNIKTSEERRLTFC 220

Sea_lamprey            IRCAESGEHYEIMLLHFLQEHL 946
Western_clawed_frog    IRCPESGEHYEITLLHFLQEHL 856
Humans                 IRCPESGEHYEVTLLHFLQEYL 892
Rhesus_macaque         IRCPESGEHYEVTLLHFLQEYL 892
House_mouse            IRCRESGEHYEVTLLHFLQEHL 862
Common_wall_lizard     IRCPESGEHYEITLLHFLQEYL 889
Green_sea_turtle       IRCPESGEHYEITLLHFLQEYL 892
Chicken                IRCPESGEHYEITLLHFLQEYL 931
Saltwater_crocodile    IRCPESGEHYEITLLHFLQEYL 892
Monterrey_platyfish    IRCPESGEHYEIMLLHFLQQYL 893
Zebrafish              IRCPESGEHYEIMLLYFLQQHL 885
Electric_eel           IRCPESGEHYEIMLLYFLQQHL 885
Channel_catfish        IRCPESGEHYEIMLLHFLQQHL 886
```

DPP8 Multiple Sequence Alignment

```
West_African_lungfish  IWLSNIEAGEERRLTFVHEGLP 240
Thorny_skate           LWISNIESGEERRLTFAHKGLP 297
Tropical_clawed_frog   LWIGNVETGEERRLTFVHKDLA 247
Common_toad            LWVANIESGEERRLTFVHKDLV 243
Common_wall_lizard     IWISNLATKEERRLTFVHKEFA 259
House_mouse            IWISNLVTREERRITYVHNELA 251
Humans                 IWISNIVTREERRLTYVHNELA 241
Rhesus_monkey          IWISNIVTREERRLTYVHNELA 250
Green_sea_turtle       IWISNILTREERRLTFVHNELA 241
Painted_turtle         IWISNILTREERRLTFVHNELA 241
Chicken                IWISNIETREERRLTFVHNELA 241
Saltwater_crocodile    IWISNLETREERRLTFVHNELA 242

West_African_lungfish  HSLRVPESGEHYELHLLFYLQE 869
Thorny_skate           HSIRVPESGEHYELYLLYYLQE 926
Tropical_clawed_frog   HSIRVPESGEHYELHLLYYLQE 876
Common_toad            HSIRVPESGEHYELHLLYYLQE 872
Common_wall_lizard     HSIRVPESGEHYELHLLYYLQE 888
House_mouse            HSIRVPESGEHYELHLLHYLQE 880
Humans                 HSIRVPESGEHYELHLLHYLQE 870
Rhesus_monkey          HSIRVPESGEHYELHLLHYLQE 879
Green_sea_turtle       HSIRVPESGEHYELHLLHYLQE 900
Painted_turtle         HSIRVPESGEHYELHLLHYLQE 870
Chicken                HSIRVPESGEHYELHLLYYLQE 870
Saltwater_crocodile    HSIRVPESGEHYELHLLYYLQE 871
```

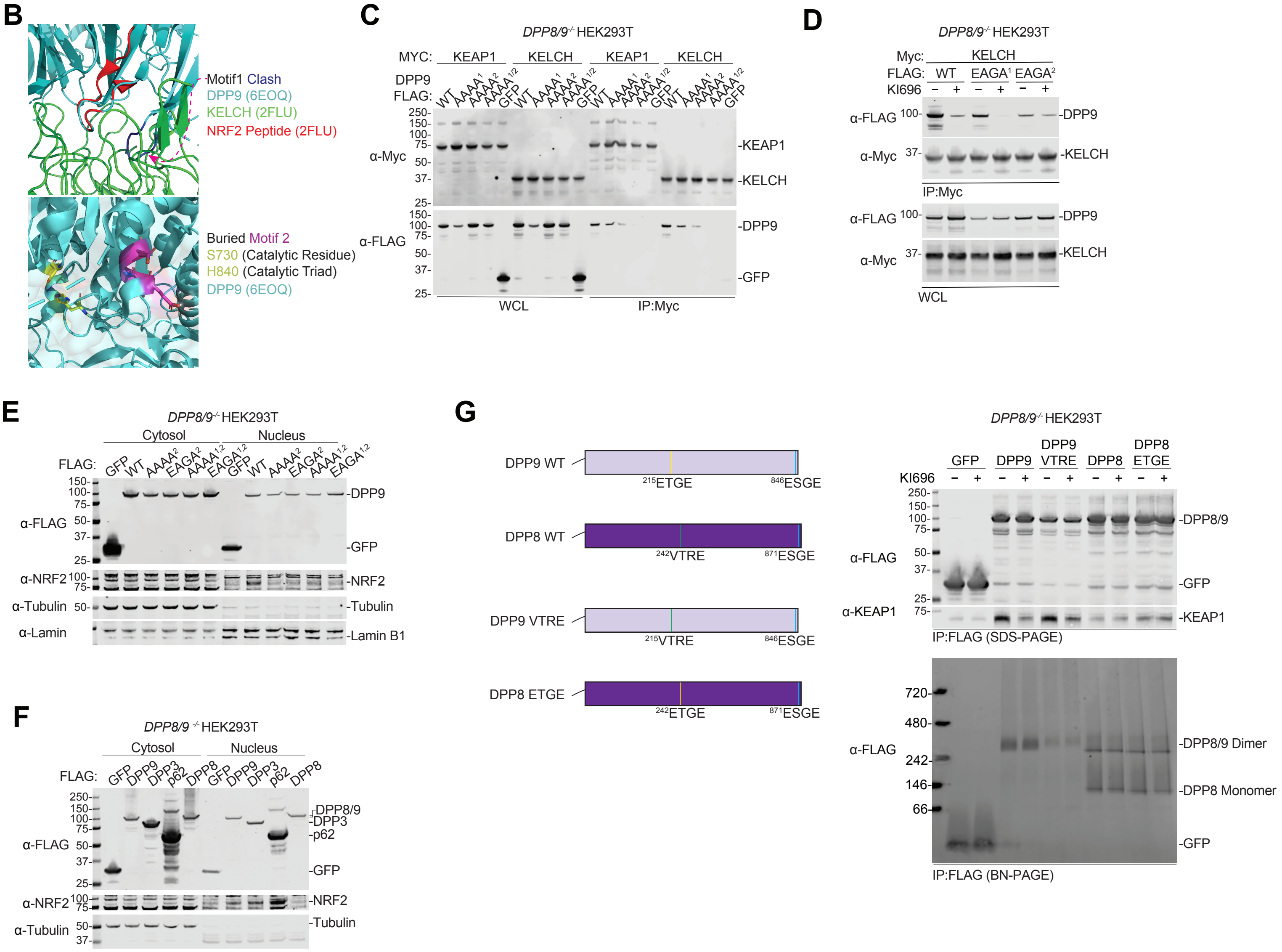

Fig S2. DPP9 ESGE Motif 2 is conserved and mediates KEAP1 binding. (**A**) Multiple sequence alignment of the regions surrounding Motif 1 (top) and Motif 2 (bottom) in DPP9 (left) and DPP8 (right) from the indicated species. The conserved Motifs are highlighted pink, and the non-conserved Motifs are highlighted blue. Phylogenic trees were created using Clustal Omega. (**B**) Overlay of the indicated PDB structures and Motif 1 (top) and Motif 2 (bottom). (**C-G**) The indicated WT and mutant proteins were transiently expressed in *DPP8/9*$^{-/-}$ HEK 293T cells for 48 h before anti-Myc (C,D) or nuclear fractionation (E,F) or anti-FLAG IP (G) followed by immunoblotting analyses. KI696 (5 μM) was added to the indicated samples at the time of transfection. In **G**, the DPP8 WT, DPP9 WT, DPP9 VTRE, and DPP8 ETGE mutant proteins are diagramed, and the bottom immunoblot is BN-PAGE. All data, including immunoblots, are representative of three or more independent experiments.

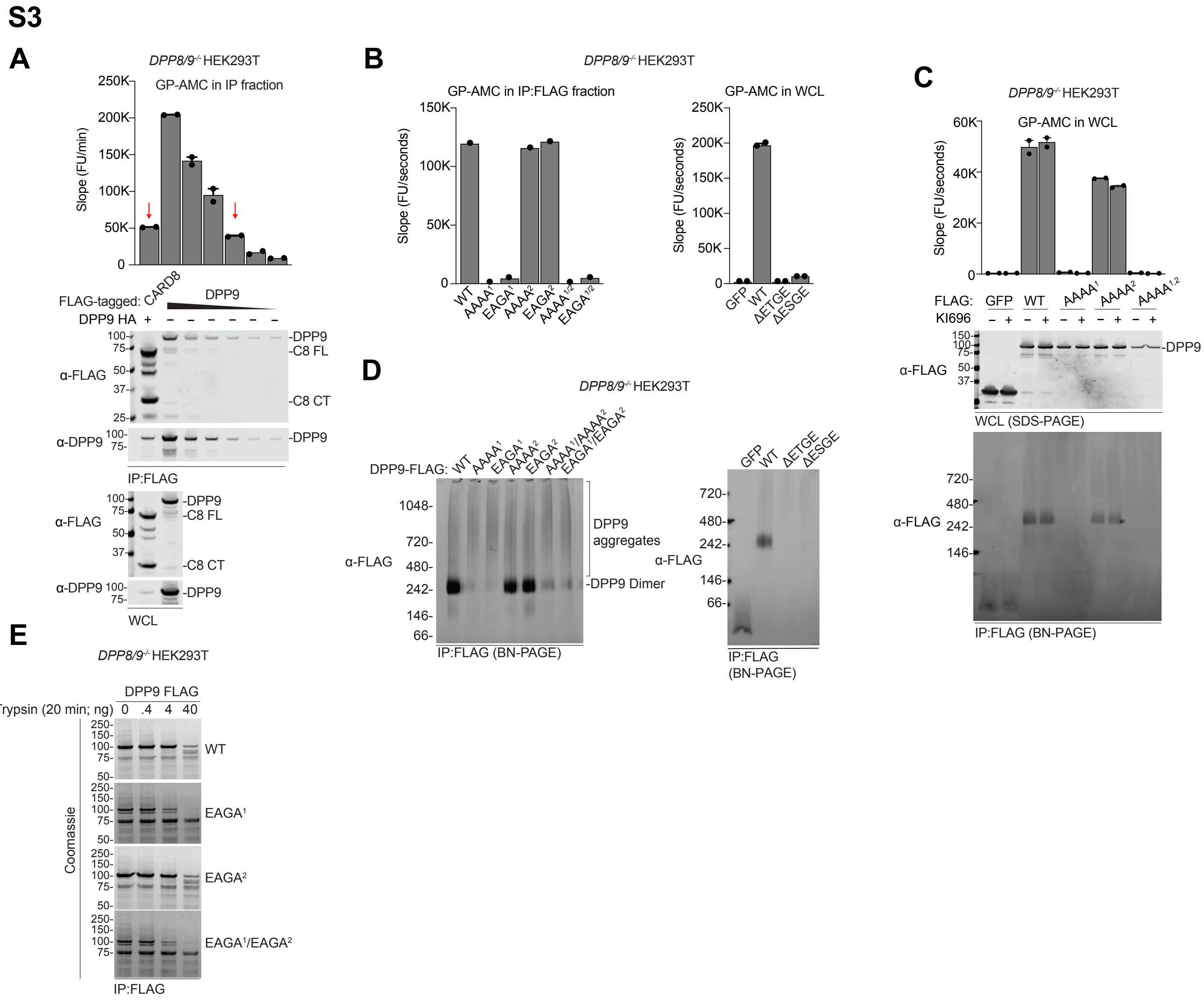
S3
A
DPP8/9-/- HEK293T
GP-AMC in IP fraction
Slope (FU/min)
FLAG-tagged: CARD8
DPP9
DPP9 HA
α-FLAG
α-DPP9
IP:FLAG
WCL
-DPP9
-C8 FL
-C8 CT
B
DPP8/9-/- HEK293T
GP-AMC in IP:FLAG fraction
GP-AMC in WCL
Slope (FU/seconds)
WT
AAAA¹
EAGA¹
AAAA²
EAGA²
AAAA¹/²
EAGA¹/²
GFP
ΔETGE
ΔESGE
C
DPP8/9-/- HEK293T
GP-AMC in WCL
FLAG:
KI696
AAAA¹,²
WCL (SDS-PAGE)
IP:FLAG (BN-PAGE)
D
DPP8/9-/- HEK293T
DPP9-FLAG:
AAAA¹/AAAA²
EAGA¹/EAGA²
DPP9 aggregates
-DPP9 Dimer
E
DPP8/9-/- HEK293T
DPP9 FLAG
Trypsin (20 min; ng)
Coomassie
EAGA¹/EAGA²

Fig S3. Some DPP9 mutants are intrinsically misfolded. (**A**) FLAG-tagged DPP9 was expressed alone, and HA-tagged DPP9 was co-expressed with FLAG-tagged CAR8 in HEK 293T cells. The resulting lysates were subjected to anti-FLAG IP, and the protein levels and DPP9 activity in the eluates were assessed by immunoblotting and GP-AMC reporter assays, respectively. The DPP9-FLAG eluate was assessed in a five-point, two-fold dilution series to normalize the free DPP9 protein levels to the CARD8-bound DPP9 protein levels (red arrows indicate comparable levels). (**B-D**) The indicated WT and mutant DPP9 proteins were transiently expressed in *DPP8/9*$^{-/-}$ HEK 293T cells. KI696 (5 μM) was added to the indicated samples at the time of transfection. After 48 h, lysates were harvested and subjected to anti-FLAG IP. DPP9 activity in lysates or IP fraction was assessed using GP-AMC assays. The indicated samples were also separated by SDS-PAGE or BN-PAGE, as indicated, and analyzed by immunoblotting. The respective SDS-PAGE immunoblots for the samples in **D** are shown in **Figure 1G** and **H**. (**E**) The indicated DPP9 proteins were expressed in in *DPP8/9*$^{-/-}$ HEK 293T cells and IPed using anti-FLAG beads. The eluates were treated with trypsin as indicated and analyzed by Coomassie staining. GP-AMC data are means ± SEM. All data, including immunoblots, are representative of three or more independent experiments.

S4

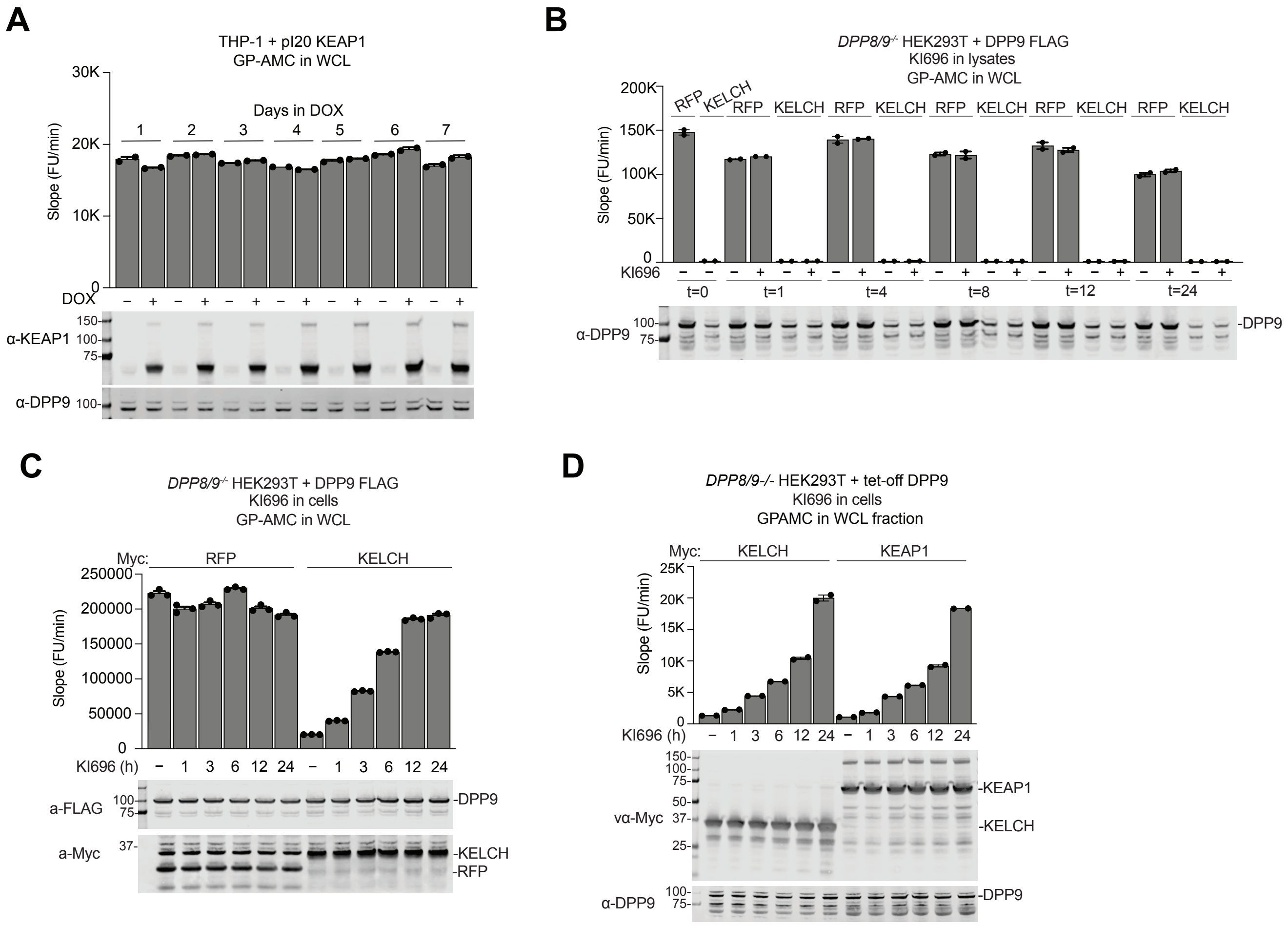
A
THP-1 + pI20 KEAP1
GP-AMC in WCL
Days in DOX
1 2 3 4 5 6 7
Slope (FU/min)
30K 20K 10K 0
DOX – + – + – + – + – + – + – +
α-KEAP1
150 100 75
α-DPP9
100
B
DPP8/9-/- HEK293T + DPP9 FLAG
KI696 in lysates
GP-AMC in WCL
RFP KELCH RFP KELCH RFP KELCH RFP KELCH RFP KELCH RFP KELCH
Slope (FU/min)
200K 150K 100K 50K 0
KI696
t=0 t=1 t=4 t=8 t=12 t=24
α-DPP9
100 75
-DPP9
C
DPP8/9-/- HEK293T + DPP9 FLAG
KI696 in cells
GP-AMC in WCL
Myc: RFP KELCH
Slope (FU/min)
250000 200000 150000 100000 50000 0
KI696 (h) – 1 3 6 12 24 – 1 3 6 12 24
a-FLAG
100 75
-DPP9
a-Myc
37-
-KELCH
-RFP
D
DPP8/9-/- HEK293T + tet-off DPP9
KI696 in cells
GPAMC in WCL fraction
Myc: KELCH KEAP1
Slope (FU/min)
25K 20K 15K 10K 5K 0
KI696 (h) – 1 3 6 12 24 – 1 3 6 12 24
vα-Myc
150- 100- 75- 50- 37- 25-
-KEAP1
-KELCH
α-DPP9
100- 75-
-DPP9

Fig S4. DPP9 requires additional cellular factors to refold upon dissociation from KELCH. (**A**) THP-1 cells with a stably integrated, HA-tagged, and DOX-inducible KEAP1 construct were treated with DOX (100 ng/mL) for the indicated number of days. DPP9 activity in cells and KEAP1 and DPP9 protein levels in lysates were assessed using GP-AMC reporter assays and immunoblotting, respectively. (**B-D**) DPP9 was transiently expressed in *DPP8/9*$^{-/-}$ HEK 293T cells under a constitutive (**B,C**) or DOX-off vector (**D**) along with RFP, KELCH or KEAP1. KI696 was added to the indicated samples after lysis (25 μM, **B**) or in cells (5 μM, **C,D**). GP-AMC data are means ± SEM. All data, including immunoblots, are representative of three or more independent experiments.

S5

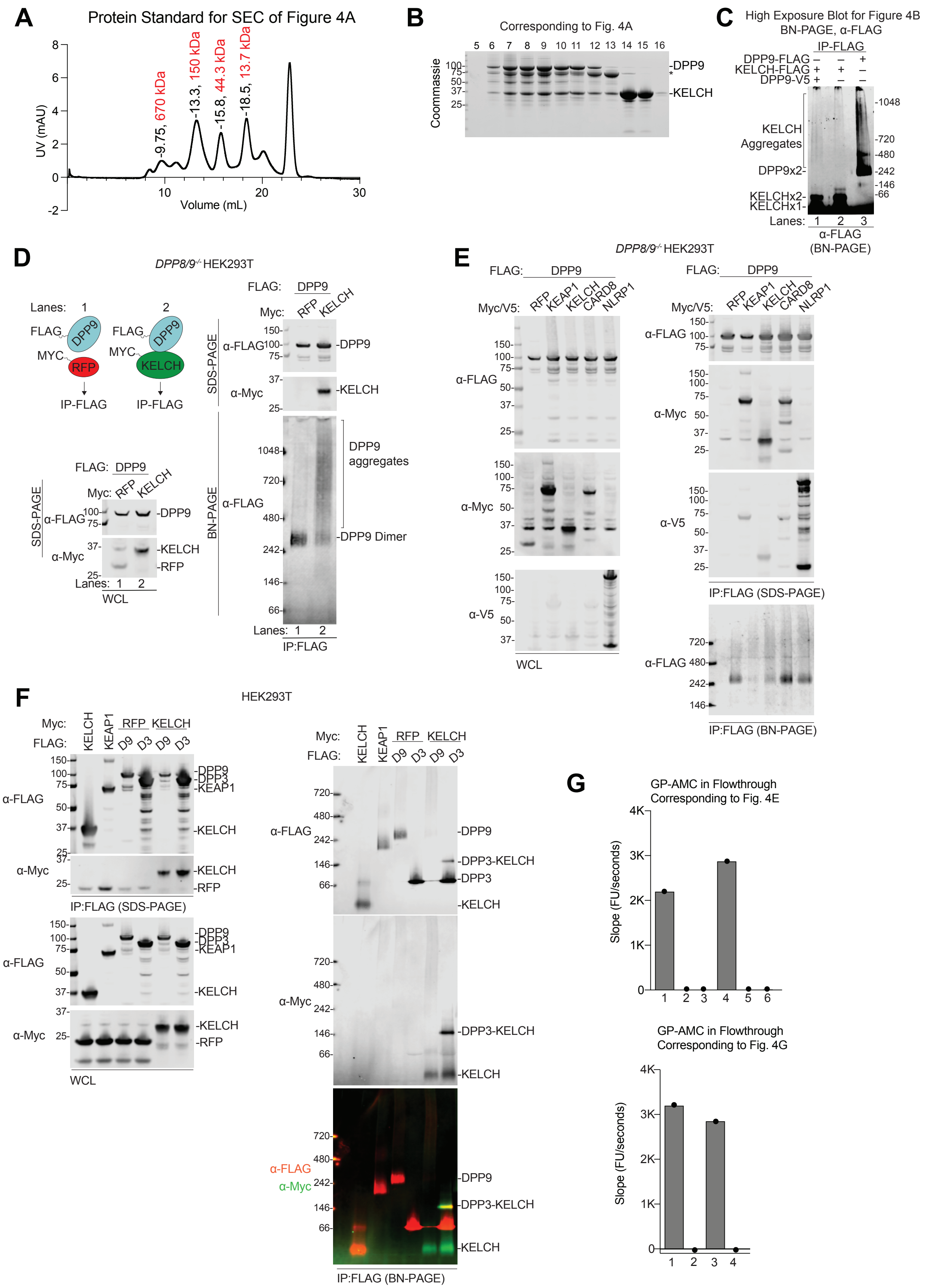
A
Protein Standard for SEC of Figure 4A
-9.75, 670 kDa
-13.3, 150 kDa
-15.8, 44.3 kDa
-18.5, 13.7 kDa
UV (mAU)
Volume (mL)
B
Corresponding to Fig. 4A
Coommassie
-DPP9
-KELCH
C
High Exposure Blot for Figure 4B
BN-PAGE, α-FLAG
IP-FLAG
DPP9-FLAG
KELCH-FLAG
DPP9-V5
KELCH Aggregates
DPP9x2-
KELCHx2-
KELCHx1-
Lanes:
α-FLAG
(BN-PAGE)
D
DPP8/9-/- HEK293T
Lanes:
FLAG
MYC
DPP9
RFP
KELCH
IP-FLAG
SDS-PAGE
BN-PAGE
α-FLAG
α-Myc
-DPP9
-KELCH
-RFP
DPP9 aggregates
-DPP9 Dimer
WCL
IP:FLAG
E
DPP8/9-/- HEK293T
FLAG:
DPP9
Myc/V5:
RFP
KEAP1
KELCH
CARD8
NLRP1
α-FLAG
α-Myc
α-V5
WCL
IP:FLAG (SDS-PAGE)
IP:FLAG (BN-PAGE)
F
HEK293T
Myc:
FLAG:
KELCH
KEAP1
RFP
D9
D3
-DPP9
-DPP3
-KEAP1
-KELCH
-RFP
-DPP3-KELCH
α-FLAG
α-Myc
IP:FLAG (SDS-PAGE)
WCL
IP:FLAG (BN-PAGE)
G
GP-AMC in Flowthrough
Corresponding to Fig. 4E
GP-AMC in Flowthrough
Corresponding to Fig. 4G
Slope (FU/seconds)

Fig. S5. DPP9 adopts an unfolded conformation only in the presence of KEAP1. (**A**) A protein standard was run through the SEC column, and the molecular weights were assigned to the respective elution fractions. (**B**) The fractions from Main Figure 3A were analyzed by Coomassie staining. (**C**) Higher exposure blot corresponding to Main Figure 4B. (**D-F**) The indicated proteins were transiently expressed in *DPP8/9*$^{-/-}$ (**D,E**) or WT (**F**) HEK 293T cells and subjected to IP FLAG purification. (**G**) GP-AMC cleavage activity of each FT fraction. Top panel corresponds to 4E, bottom panel corresponds to 4G. The indicated fractions were subjected to immunoblotting. All data, including immunoblots, are representative of three or more independent experiments.

S6

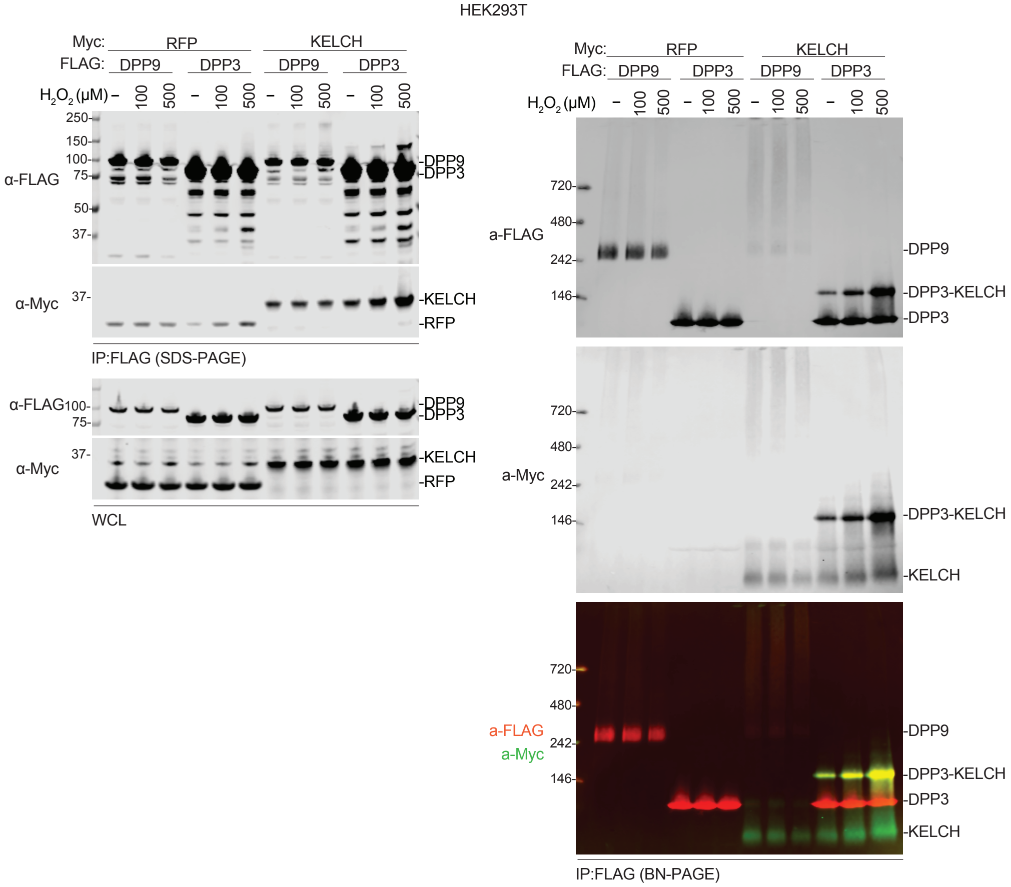
HEK293T
Myc: RFP KELCH
FLAG: DPP9 DPP3 DPP9 DPP3
H_2O_2 (µM) – 100 500 – 100 500 – 100 500 – 100 500
α-FLAG
250-
150-
100-
75-
50-
37-
-DPP9
-DPP3
α-Myc
37-
-KELCH
-RFP
IP:FLAG (SDS-PAGE)
α-FLAG
100-
75-
-DPP9
-DPP3
α-Myc
37-
-KELCH
-RFP
WCL
a-FLAG
720-
480-
242-
146-
-DPP9
-DPP3-KELCH
-DPP3
a-Myc
-DPP3-KELCH
-KELCH
a-FLAG
a-Myc
-DPP9
-DPP3-KELCH
-DPP3
-KELCH
IP:FLAG (BN-PAGE)

Fig. S6. H_2O_2 does not impact the DPP9-KEAP1 interaction. The indicated proteins were transiently expressed in HEK 293T WT cells. After 48 h, cells were treated with the indicated concentrations of H_2O_2 for 3h before lysates were harvested. The FLAG-tagged proteins were purified using anti-FLAG agarose beads, and the indicated fractions were analyzed by immunoblotting. GP-AMC data are means ± SEM. All data, including immunoblots, are representative of three or more independent experiments.

Table S1

Rank	Protein Name	Abundance Ratio (DPP9 WT/GFP)	Rank	Protein Name	Abundance Ratio (DPP9 S730A/GFP)
1	DPP9	3.900	1	HSPA6	3.252
2	MAGED1	2.442	2	MAGED1	3.184
3	BAG2	2.396	3	DPP9	3.059
4	KEAP1	2.329	4	DNAJB1	3.059
5	OTUD4	2.000	5	BAG2	2.921
6	NTPCR	1.938	6	DNAJA1	2.868
7	DNAJA1	1.916	7	DNAJB4	2.847
8	ACT	1.911	8	HSPA1B	2.837
9	RAP1B	1.873	9	BAG3	2.727
10	PSTPIP1	1.826	10	GET3	2.662
11	TIMM50	1.776	11	DNAJA2	2.490
12	NDUFA4	1.742	12	RHBDD2	2.411
13	AHCY	1.690	13	NDUFA4	2.351
14	PHKG2	1.667	14	NTPCR	2.344
15	SLC25A5	1.653	15	KEAP1	2.300
16	RIOK1	1.653	16	SLC25A5	2.279
17	COPRS	1.644	17	OTUD4	2.258
18	ECHDC1	1.639	18	FAF2	2.252
19	DNAJA2	1.635	19	SUMO4	2.245
20	DNAJA3	1.635	20	SUMO3	2.238

Table S1. List of the DPP9 binding partners identified in quantitative Mass Spectrometry analyses.
